# 3D Biomimetic Calcified Cartilaginous Callus that Induces Type H Vessels Formation and Osteoclastogenesis

**DOI:** 10.1002/advs.202207089

**Published:** 2023-03-31

**Authors:** Minglong Qiu, Changwei Li, Zhengwei Cai, Cuidi Li, Kai Yang, Nijiati Tulufu, Bo Chen, Liang Cheng, Chengyu Zhuang, Zhihong Liu, Jin Qi, Wenguo Cui, Lianfu Deng

**Affiliations:** ^1^ Department of Orthopaedics Shanghai Key Laboratory for Prevention and Treatment of Bone and Joint Diseases Shanghai Institute of Traumatology and Orthopaedics Ruijin Hospital Shanghai Jiao Tong University School of Medicine 197 Ruijin 2nd Road Shanghai 200025 P. R. China

**Keywords:** 3D printing, biomimetic calcified cartilaginous callus, osteoclastogenesis, osteogenesis, type H vessels

## Abstract

The formation of a calcified cartilaginous callus (CACC) is crucial during bone repair. CACC can stimulate the invasion of type H vessels into the callus to couple angiogenesis and osteogenesis, induce osteoclastogenesis to resorb the calcified matrix, and promote osteoclast secretion of factors to enhance osteogenesis, ultimately achieving the replacement of cartilage with bone. In this study, a porous polycaprolactone/hydroxyapatite‐iminodiacetic acid‐deferoxamine (PCL/HA‐SF‐DFO) 3D biomimetic CACC is developed using 3D printing. The porous structure can mimic the pores formed by the matrix metalloproteinase degradation of the cartilaginous matrix, HA‐containing PCL can mimic the calcified cartilaginous matrix, and SF anchors DFO onto HA for the slow release of DFO. The in vitro results show that the scaffold significantly enhances angiogenesis, promotes osteoclastogenesis and resorption by osteoclasts, and enhances the osteogenic differentiation of bone marrow stromal stem cells by promoting collagen triple helix repeat‐containing 1 expression by osteoclasts. The in vivo results show that the scaffold significantly promotes type H vessels formation and the expression of coupling factors to promote osteogenesis, ultimately enhancing the regeneration of large‐segment bone defects in rats and preventing dislodging of the internal fixation screw. In conclusion, the scaffold inspired by biological bone repair processes effectively promotes bone regeneration.

## Graphical abstract

Based on the natural bone repair process, a 3D biomimetic calcified cartilaginous callus (PHS) is prepared using calcium ion chelation and 3D printing technology to promote the regeneration of large‐segment bone defects. PHS can promote both type H vessels formation and osteoclast formation and accelerate bone regeneration by facilitating the expression of osteogenesis‐promoting coupling factors.

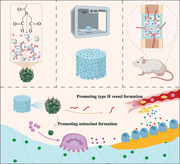

## 1 Introduction

The regeneration of large‐segment bone defects caused by infection, trauma, and tumors remains a substantial clinical challenge owing to the lack of normal bone repair processes, leading to delayed healing or even nonhealing.^[^ ^1^ ^]^ Normal bone healing is similar to bone development and involves multiple physiological events, including hematoma formation and inflammatory responses, recruitment of progenitor cells to produce cartilage, revascularization and calcification, and bone remodeling.^[^ ^2^ ^]^ After cartilage formation, chondrocytes undergo hypertrophy and calcify the extracellular matrix to form calcified cartilaginous callus.^[^ ^3^ ^]^ The formation of a calcified cartilaginous callus provides a relatively stable bridge for bone regeneration.^[^ ^4^ ^]^ Physiologically, on the one hand, matrix metalloproteinases secreted by hypertrophic chondrocytes within the calcified cartilaginous callus degrade the cartilaginous callus to form pores and secrete vascular endothelial growth factor (VEGF) to promote vascular invasion into the pores. Vascular invasion brings cells and nutrients required for bone regeneration to the defect site, and specific vascular subtypes also couple angiogenesis and osteogenesis to promote bone regeneration.^[^ ^5^ ^]^ On the other hand, the calcified cartilaginous matrix stimulates the formation of osteoclasts and subsequent resorption. It induces the expression of various factors to promote osteogenesis and completely replaces the cartilaginous callus with a bony callus.^[^ ^6^ ^]^ However, large‐segment bone defects cannot form calcified cartilaginous calluses, making their regeneration challenging. Therefore, reconstructing a calcified cartilaginous callus is critical for regenerating large‐segment bone defects.

Osteogenesis and angiogenesis are closely related to bone regeneration and remodeling in the mammalian skeletal system.^[^ ^7^ ^]^ There are two types of blood vessels in the bone: type H and type L. Type H vascular endothelial cells express high levels of CD31 and endothelial mucin (EMCN).^[^ ^5^ ^]^ Type H vessels are organized into straight columns interconnecting at their distal ends.^[^ ^5^ ^]^ They are distributed near the growth plate and in trabecular and cortical bone along the periosteal and endosteal surfaces. In contrast, type L vessels express low levels of CD31 and EMCN and form a dense, highly branched capillary network in the marrow lumen of the backbone.^[^ ^5^ ^]^ Oxygen rich blood flows from the arteries and distal arterioles and connects directly to type H vessels, and then continues to the type L sinusoidal network at the interface between the metaphysis and diaphysis, eventually draining into the central vein.^[^ ^7^ ^]^ It was confirmed that type H vessels are the main vessels associated with osteogenesis and that most osteoprogenitor cells (≈70% of Osterix+ osteoprogenitor cells) selectively localize near type H vessels.^[^ ^5^ ^]^ Type H vessels actively direct bone formation by producing factors that stimulate the proliferation and differentiation of osteoprogenitor cells in the bone marrow.^[^ ^5^, ^8^ ^]^ During bone repair, as calcified cartilaginous callus form, hypertrophic chondrocytes secrete VEGF to induce type H vessels, which promote osteogenesis by inducing osteoprogenitor cells to colonize in their vicinity and by secreting various growth factors, including transforming growth factor *β* (TGF‐*β*), platelet‐derived growth factor (PDGF), and fibroblast growth factor 1 (FGF‐1).^[^ ^5^, ^7^ ^]^ Hypoxia‐inducible factors (HIFs) have been reported to be the main factors promoting type H vessels formation.^[^ ^5^ ^]^ We and other studies have confirmed that activation of the hypoxia‐inducible factor‐1α (HIF‐1*α*) pathway stimulates the expression of VEGF and osteogenesis‐related proteins to accelerate bone repair.^[^ ^9^ ^]^ Thus, the beneficial effects of the hypoxia/HIF‐1 pathway activation on angiogenesis and bone formation have prompted researchers to search for hypoxia mimetic agents (HMAs) that activate the hypoxia pathway.^[^ ^10^ ^]^

Deferoxamine (DFO), an FDA‐approved iron chelator used to treat iron toxicity or iron overload, activates the HIF‐1 pathway and is widely used as an HMA under normoxic conditions.^[^ ^11^ ^]^ Our previous studies have shown that DFO or DFO‐loaded scaffolds increase the amount of neovascularization and new bone formation in the bone callus tissue and promote bone healing.^[^ ^9^, ^12^ ^]^ It was recently found that DFO promote type H vessels formation by stabilizing HIF‐1*α* in aging mice, which accelerates bone repair process.^[^ ^5^, ^13^ ^]^ However, DFO is water‐soluble, and although DFO‐loaded scaffolds were prepared by 3D printing and material surface modification plasma layer self‐assembly technologies, they could not prevent the rapid release of DFO from the scaffold and the “sudden release” (up to 90% release at 48 h) after implantation.^[^ ^11^ ^]^ Therefore, we used the calcium chelating agent iminodiacetic acid ((IDA, NH (CH _2_ COOH) _2_), SF) as a lead compound of DFO to develop bone‐seeking SF‐DFO; the chemical modification of SF did not alter the performance of DFO‐induced HIFs; systemic high‐dose application did not lead to DFO‐induced diffuse liver, spleen, kidney, or other organ inflammatory responses, inflammatory infiltration, granular cellular degeneration, interfacial hepatitis, or hepatocyte debris necrosis.^[^ ^14^ ^]^ Therefore, applying SF‐DFO in combination with calcified biomaterials to construct a biomimetic calcified cartilaginous callus that induces type H vessels has unique advantages in regenerating large‐segment bone defects.

Hydroxyapatite (HA), the main inorganic component of the bone, is one of the most widely used calcifying biomaterials.^[^ ^15^ ^]^ Calcium‐containing matrix materials stimulate osteoclast formation and differentiation.^[^ ^16^ ^]^ Our previous study also showed calcium‐containing *β*‐tricalcium phosphate enhances osteoclast differentiation.^[^ ^17^ ^]^ Osteoclasts are involved in all steps of the bone repair process, including the resorption of necrotic bone fragments during the early stages of inflammation, resorption of the calcified cartilaginous callus, and coupling to osteogenesis in the middle stages, remodeling of the hard bone callus during the bone remodeling phase according to Wolff's law, and restoration of the bone to a size similar to that before the injury.^[^ ^18^ ^]^ The microenvironment created via the resorption of biological material by osteoclasts is more conducive to osteoblast osteogenesis than to physical and chemical degradation.^[^ ^16a^ ^]^ In addition, osteoclasts secrete collagen triple helix repeat‐containing 1 (CTHRC1) to promote the osteogenic differentiation of bone marrow stromal stem cells (BMSCs) when stimulated by calcium‐containing biomaterials, such as HA.^[^ ^6b^ ^]^ The absorbed calcium and phosphorus also serve as raw materials for bone remodeling to facilitate bone repair.^[^ ^16^, ^19^ ^]^ Therefore, using HA as a raw material for calcification in combination with SF‐DFO to induce type H vessels formation will mimic the normal biological process of calcified cartilaginous callus formation and subsequent replacement by bone.

**Scheme 1:**
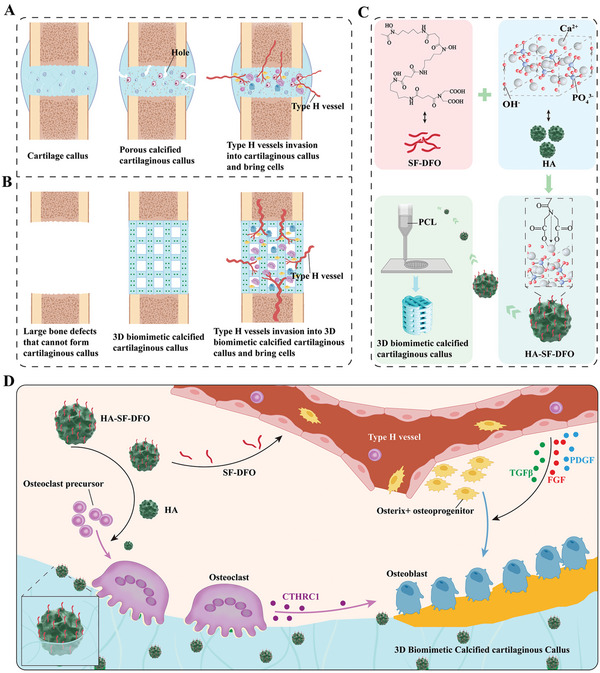
Construction of a 3D biomimetic cartilaginous callus and its mechanism of promoting the regeneration of large‐segment bone defects. A) Normal biological process of bone regeneration. B) Large‐segment bone defects cannot form calcified cartilaginous calluses or type H vessels. A 3D biomimetic calcified cartilaginous callus with coupled type H vessels formation was constructed to promote the regeneration of large‐segment bone defects. C) Construction of 3D biomimetic calcified cartilaginous callus. D) 3D biomimetic calcified cartilaginous callus promotes osteoclast and type H vessels formation to promote osteogenesis. (Scheme 1 was created with BioRender.com.)

3D printing technology allows the construction of scaffolds with set dimensions and structures and the creation of composites of multiple materials.^[^ ^20^ ^]^ The construction of a porous 3D biomimetic calcified cartilaginous callus that matches the size of large bone defects and facilitates vascularization via 3D printing is particularly important for bone regeneration. In this study, SF‐DFO was chelated with HA to form HA‐SF‐DFO, a HA microparticle that promotes osteoclast differentiation and type H vessels induction. HA‐SF‐DFO was compounded with polycaprolactone (PCL) to construct a porous, long‐term, type H vessel‐inducing PCL/HA‐SF‐DFO 3D biomimetic calcified cartilaginous callus (PHS) using fused 3D bioprinting technology (**Scheme** **1**C). In vitro, the ability of PHS to promote angiogenesis was assessed by human umbilical vein endothelial cell (HUVEC) tubule formation assays and chick embryo chorioallantoic membrane (CAM) angiogenesis assays; the potential of PHS to promote osteoclast formation and resorption by osteoclasts was assessed by osteoclast induction and resorption assays; and the ability of PHS to promote osteoclast CTHRC1 expression and coupled osteogenesis was verified by small interfering RNA knockdown and osteoclast‐BMSC coculture techniques. In vivo, we evaluated the ability of PHS to promote type H angiogenesis and coupled osteogenesis, verified the ability of PHS to promote the regeneration of large‐segment bone defects in a rat femoral large‐segment bone defect model, and further verified the ability of PHS to promote the osseointegration of internal fixation screws in an aseptic loosening model.

## 2 Results and Discussion

### 2.1 Fabrication and Characterization of PHS

**Figure 1:**
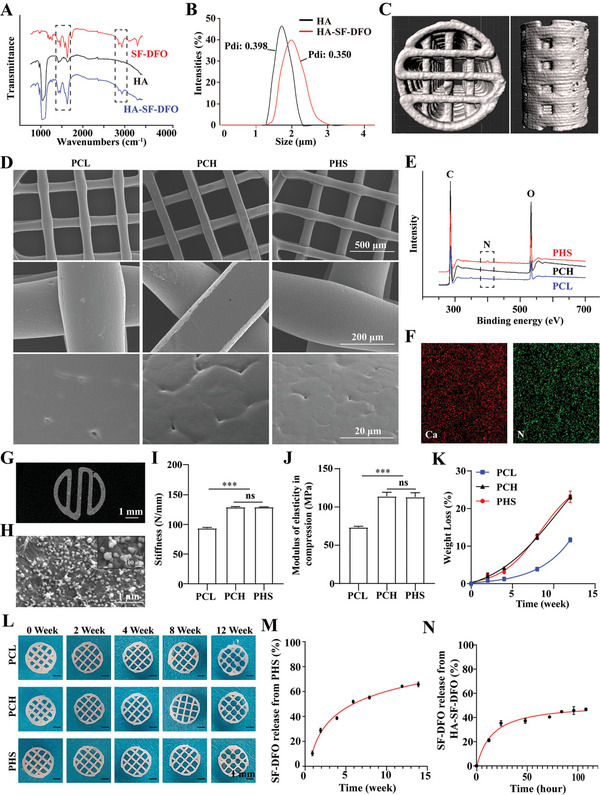
Fabrication and characterization of PHS. A) Infrared spectra of SF‐DFO, HA, and HA‐SF‐DFO. The black dashed box shows the characteristic absorption peaks of SF‐DFO. B) Particle size distribution of HA and HA‐SF‐DFO. C) 3D structure of PHS reconstructed by Imaris after micro‐CT scanning. D) SEM images of PCL, PCH, and PHS. E) Photoelectron spectra of PCL, PCH, and PHS. The black dashed boxes show the characteristic nitrogen elements of SF‐DFO inside PHS. F) SEM‐EDS shows the distribution of calcium and nitrogen within PHS. G) Micro‐CT scan shows the distribution of HA within PHS. H) SEM image shows the distribution of HA in the PHS. I,J) Stiffness I) and compressive modulus of elasticity J) of PCL, PCH, and PHS, *n* = 3. K) Weight loss curves of PCL, PCH, and PHS in PBS, *n* = 3. L) Photograph of the scaffold degrading over time. M) Curve of the percentage of SF‐DFO released from PHS, *n* = 3. N) Curve of the percentage of SF‐DFO released from HA‐SF‐DFO, *n* = 3. All data are expressed as the mean ± SD. For I,J), statistical analysis was performed using one‐way ANOVA with Tukey's multiple comparison tests. ns, no significant difference. ***p < 0.001. HA, hydroxyapatite; HA‐SF‐DFO, hydroxyapatite‐iminodiacetic acid‐deferoxamine; PCL, polycaprolactone scaffold; PCH, PCL/HA scaffold; PHS, PCL/HA‐SF‐DFO 3D biomimetic calcified cartilaginous callus.

Because SF‐DFO is fat‐soluble, it was first dissolved in dimethyl sulfoxide to form a saturated solution. HA powder was then added to the solution, and HA‐SF‐DFO was synthesized by chelating SF in SF‐DFO with calcium ions in HA (Scheme 1C; and Figure S1A, Supporting Information). By comparing the characteristic peaks of the infrared spectra of the HA, SF‐DFO, and HA‐SF‐DFO powders, it was confirmed that HA was successfully conjugated with SF‐DFO (**Figure** **1**A). The z‐average particle size of HA‐SF‐DFO was larger than that of HA, suggesting that HA was conjugated with SF‐DFO (Figure 1B). We further investigated the efficacy of SF‐DFO in binding to HA. We added 50 mg of HA to 1 mL of different concentrations of SF‐DFO solution to study its binding efficacy. The results showed that ≈1.25 ± 0.05 mg SF‐DFO was bound per 50 mg HA. At low concentrations, SF‐DFO in solution was completely bound; when saturation binding was reached, the concentration of SF‐DFO did not seem to affect the binding efficacy (Figure S1G, Supporting Information). The binding of SF‐DFO to HA was completed quickly, and prolongation of the time (0.5–12 h) did not increase the binding of SF‐DFO to HA (Figure S1H, Supporting Information).

PCL has a low melting point (which is favorable for molding during manufacturing) and has suitable properties for bone tissue regeneration.^[^ ^21^ ^]^ They can be fabricated into desired porous structures using 3D printing.^[^ ^11^ ^]^ We constructed a porous PHS as a bone repair scaffold material using a fused 3D printing technique. We set the printed filament diameter as 200 µm and the filament pitch as 400 µm. Consistent with previous findings, 100–1200 µm is ideal for vascularized pores.^[^ ^22^ ^]^ The liquefier temperature was set to 90 °C.^[^ ^23^ ^]^ The PCL/HA scaffold (PCH) exhibited the best mechanical properties when the mass percentage of HA was 20%.^[^ ^24^ ^]^ Since the mass ratio of SF‐DFO was extremely low (0.50 ± 0.02%), we used 20% and 80% mass percentages of HA‐SF‐DFO and PCL, respectively, to fabricate PHS scaffolds in this study. The PHS was fabricated using a 0/90° laminated pattern of cellular lattice structures and continuous contour filaments to ensure a 3D porous spatial structure that mimics a calcified cartilaginous callus (Figure 1C). Cross‐sectional scanning electron microscopy (SEM) images showed the microstructural features of the PHS (Figure 1D). Video S1 (Supporting Information) shows the dynamic 3D spatial structure of the PHS.

Verify the presence and distribution of SF‐DFO within the PHS. We first analyzed the elemental composition of PHS by X‐ray photoelectron spectroscopy (XPS), and the characteristic N‐nitrogen of SF‐DFO suggested that SF‐DFO was successfully “anchored” inside PHS (Figure 1E). Further analysis of Ca and N by SEM‐energy dispersive spectrometry (SEM‐EDS) showed a uniform distribution of HA and SF‐DFO within PHS (Figure 1F). Figure S1D,F (Supporting Information) shows the elemental composition and proportions within the PHS. To evaluate the calcification of the PHS, we scanned it using micro‐CT (HA was visualized, while PCL was not). The results showed that HA was uniformly distributed within the PHS and that the PHS were uniformly calcified (Figure 1G). We further observed the HA particles inside the scaffold using SEM (surface of the scaffold after cutting). The results showed that the HA particles were uniformly distributed within the PCL (Figure 1H). Compared with PCL scaffolds, the stiffness, and modulus of elasticity of PCH scaffolds increased by 37.8 ± 0.7% and 55.1 ± 0.4%, respectively, and the addition of SF‐DFO did not change the mechanical properties of the scaffold (Figure 1I,J).

Next, we evaluated the in vitro degradation properties of PHS and the release of SF‐DFO. As previously described, DFO was chelated with HA via iminodiacetic acid and anchored inside the PCL matrix (Scheme 1D). This dual‐anchoring structure allowed for the slow release of DFO. The degradation rate was assessed by placing the scaffold in phosphate buffer (PBS) and calculating the weight loss at designated time points. In agreement with previous reports,^[^ ^25^ ^]^ the addition of HA to PCL increased the degradation rate of PCL/HA owing to its increased hydrophilicity, whereas the degradation rate of PHS was consistent with that of PCH (Figure 1K,L). Remarkably, the increased degradation rate did not lead to the rapid collapse of the scaffold structure. Our results showed that by week 12, the PCH and PHS scaffolds still had distinguishable architectures (Figures 1L and 7A). Next, we detected the release of SF‐DFO. In vitro drug release assays showed a relatively rapid release of SF‐DFO from pure HA‐SF‐DFO, reaching an equilibrium value at 50 h with a percentage release of 46.7 ± 1.1% (Figure 1N). When HA‐SF‐DFO was composited with PCL, it greatly slowed the release of SF‐DFO, and the percentage of SF‐DFO released from the scaffold by week 12 was only about 64.2 ± 0.9% (Figure 1M). Thus, this method significantly slowed the release rate of the drug compared with the previous drug‐loading method.^[^ ^11^ ^]^

To assess the biocompatibility of PCH, we examined its effect on the viability and proliferation of rat BMSCs. The live/dead staining results showed that the seeded cells grew well on the PCL, PCH, and PHS surfaces, and few dead cells were observed (Figure S2A, Supporting Information). Cell viability was measured using the Cell Counting Kit‐8 (CCK‐8) on days 1, 3, and 7 after inoculation. The results showed that PHS did not significantly affect cell proliferation and that the cell proliferation rate was consistent between the groups (Figure S2C, Supporting Information). In addition, the cytoskeletal staining results showed that BMSCs adhered to and extended well on the surfaces of PCL, PCH, and PHS (Figure S2B, Supporting Information). With the degradation of the scaffold, SF‐DFO was released from the scaffold and acted on the cells. Therefore, we examined the effects of SF‐DFO on BMSCs viability. The results showed no adverse impact of SF‐DFO on the cell viability of BMSCs, even at concentrations up to 200 µm (Figure S2D, Supporting Information). These data suggest that PHS exhibits good biocompatibility.

In conclusion, these results confirm that PHS has suitable pores to mimic the pore channels created by matrix metalloproteinase degradation of the mineralized cartilaginous matrix. PHS has uniform calcification, favorable mechanical properties, and biocompatibility and can achieve long‐term release of drugs that induce type H vessels formation. Owing to its favorable biocompatibility and mechanical properties, PHS may also load seed cells (such as periosteal cells and BMSCs) or significantly enhance its bone repair potential by filling it with drug‐loaded hydrogels.^[^ ^26^ ^]^ Previous studies have demonstrated the excellent drug‐loading potential and adjustability of hydrogels. Bioactive nanocomposite hydrogels have been used for the local elution and on‐demand simultaneous release of bioactive ions and small molecule drugs.^[^ ^27^ ^]^ Loading cells, metal ions, or small‐molecule drugs promote bone regeneration.^[^ ^28^ ^]^ However, the mechanical properties of the hydrogels are insufficient to support segmental bone defects.^[^ ^29^ ^]^ Therefore, the filling of PHS with drug‐loaded hydrogels is an ideal approach.

### 2.2 PHS Promotes Osteoclastogenesis and Resorption by Osteoclasts

**Figure 2:**
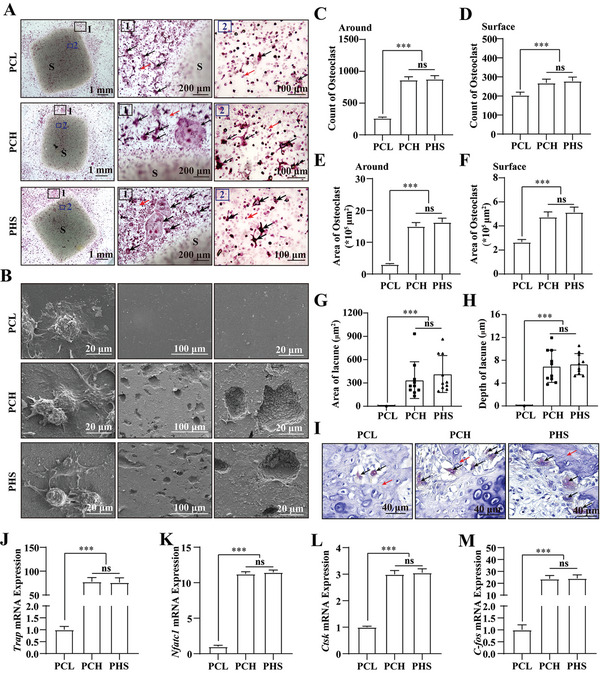
PCH and PHS promote osteoclast formation and resorption by osteoclasts. A) TRAP staining images of osteoclasts on PCL, PCH, or PHS sheets after seven days of stimulation using M‐CSF and RANKL. S, PCL, PCH, or PHS sheets. 1) Cells around the sheet. 2) Cells on the surface of the sheet. Black arrows, osteoclasts. Red arrows, osteoclast precursors. B) SEM images of osteoclasts and their resorption lacunae on the surface of the sheet. C,D) Count of osteoclasts around C) and on the surface of D) the sheet, *n* = 3. E,F) Areas of osteoclasts around E) and on the surface of F) the sheet, *n* = 3. G) Analysis of resorption area. H) Measurement of lacune depth. I) TRAP staining images of the calcified cartilaginous matrix in rats with large‐segment bone defects implanted with PCL, PCH, or PHS at 4 weeks. Black arrows, osteoclasts. Red arrows, calcified cartilaginous matrix. J–M) Statistical analysis of osteoclast formation‐related mRNA expression, including *Trap* J), *Nfatc1* K), *Ctsk* L), and *C‐fos* M), *n* = 3. All data are expressed as the mean ± SD. For C–H) and J–M), statistical analysis was performed using one‐way ANOVA with Tukey's multiple comparison tests. ns, no significant difference. ***p < 0.001.

Osteoclasts are polymorphonuclear cells formed by the fusion and differentiation of monocytes/macrophages in blood and bone marrow.^[^ ^30^ ^]^ In repairing large bone defects, bone regeneration, and remodeling processes overlap and are coordinated by communication between osteoblasts and osteoclasts.^[^ ^31^ ^]^ Osteoclasts promote bone defect healing by secreting osteogenic factors,^[^ ^32^ ^]^ and osteoclast depletion is detrimental to bone formation,^[^ ^33^ ^]^ suggesting that the osteoclastic process is essential for intact bone repair. Osteoclasts are involved in all steps of the bone repair process, including resorption of necrotic bone fragments during the early stages of inflammation, resorption of calcified cartilaginous callus and coupling to the bone during the middle stages, remodeling of hard bone callus, and restoration of the bone to a size similar to that before the injury during the bone remodeling phase.^[^ ^18^ ^]^ It has been reported that calcium ions released after HA dissolution contributes to osteoclasts’ resorptive function by acting as a second messenger to promote osteoclast differentiation.^[^ ^34^ ^]^ To verify that PHS promotes osteoclast formation, rat bone marrow‐derived monocytes were grown in culture dishes containing prefabricated PCL, PCH, or PHS sheets and cultured in a complete medium containing colony‐stimulating factor (M‐CSF) and nuclear factor *κ*B receptor activator ligand (RANKL) for seven days. Antitartrate acid phosphatase (TRAP) staining showed a significant increase in osteoclast formation around and on the surface of both PCH and PHS compared to that of PCL (**Figure** **2**A), which was confirmed by the number (Figure 2C,D) and area (Figure 2E,F) of osteoclasts around and on the surface of the sheet. In each group, the osteoclasts on the sheet surface were smaller than the surrounding osteoclasts, which can be attributed to the lower smoothness of the sheet surface compared to that of the culture dish, as a smooth surface is more beneficial for osteoclast formation.^[^ ^16a^ ^]^ PHS showed the same ability to promote osteoclast formation as PCH (Figure 2A). We further examined the expression of osteoclast formation‐related genes using quantitative real‐time polymerase chain reaction (qRT‐PCR). The results showed that the mRNA expression of *Trap* (Figure 2J), activated T‐cell nuclear factor 1 (*Nfatc1*) (Figure 2K), tissue proteinase K (*Ctsk*) (Figure 2L), and cellular oncogene ‐Fos (*C‐fos*) (Figure 2M) significantly increased in both the PCH and PHS groups. No differences were observed between the PCH and PHS groups. These results showed that HA‐containing PCH and PHS significantly promoted osteoclastogenesis and that adding SF‐DFO had no negative impact.

Biomaterial degradation involves the lysis, foreign body phagocytosis, and cell‐mediated resorption.^[^ ^35^ ^]^ After osteoclast formation, HA‐containing biomaterials can be resorbed.^[^ ^36^ ^]^ This resorption of biomaterials by osteoclasts promotes osteogenesis.^[^ ^19^ ^]^ Therefore, we next examined the resorption of PCH and PHS by osteoclasts, and SEM showed that osteoclasts formed significant resorption lacunae on both PCH and PHS (Figure 2B), whereas no resorption lacunae were evident on PCL. The area and depth of the resorption lacunae confirmed that osteoclasts could resorb PCH and PHS, and that the addition of SF‐DFO had no negative effect on osteoclast resorption, whereas PCL could not be resorbed by osteoclasts (Figure 2G,H). The formation of osteoclasts within the mineralized cartilaginous matrix was examined by TRAP staining at 4 weeks after establishing a large‐segment bone defect in the femur of rats. The results revealed a significant increase in osteoclast formation and resorption of the mineralized cartilaginous matrix in the PCH and PHS (Figure 2I) groups. In conclusion, these results confirmed that PCH and PHS containing HA significantly induced osteoclast formation and resorption by osteoclasts compared to PCL, and the addition of SF‐DFO had no negative impact.

### 2.3 PHS Promotes Osteogenic Differentiation of BMSCs by Promoting Osteoclast Expression of CTHRC1

**Figure 3:**
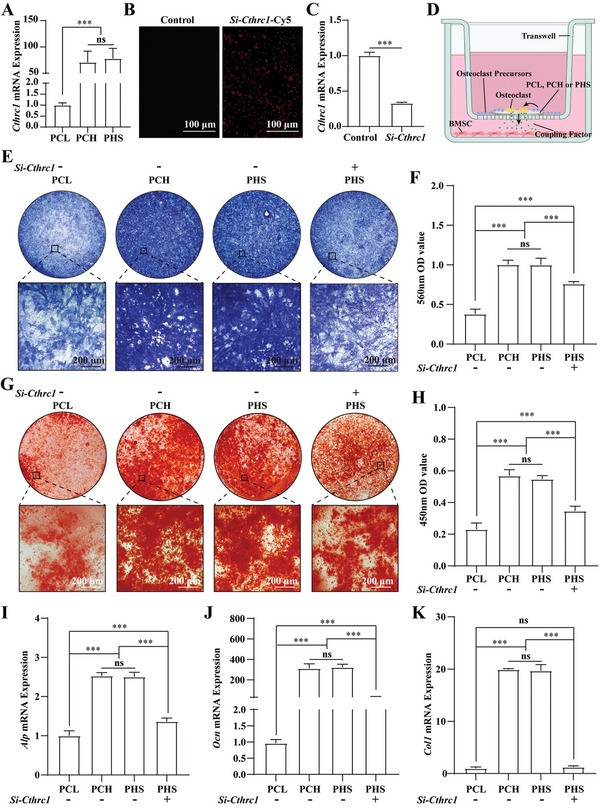
PHS promotes osteogenesis by promoting osteoclast expression of CTHRC1. A) Statistical analysis of Cthrc1 mRNA expression in osteoclasts, *n* = 3. B) Immunofluorescence image of small interfering RNA (Si‐Cthrc1) with Cy5 in osteoclast precursors. C) Expression of Cthrc1 mRNA after transfection of osteoclast precursors with Si‐Cthrc1, *n* = 3. D) Scheme of osteoclast‐BMSC coculture. E) ALP staining of BMSCs after seven days of coculture. F) Statistical analysis of the absorbance at 560 nm after ALP staining, *n* = 3. G) Alizarin red staining of BMSCs after 14 days of coculture. H) Statistical analysis of the absorbance at 450 nm after alizarin red staining, *n* = 3. I–K) Statistical analysis of Alp I), Ocn J), and Col1 K) mRNA expression in BMSCs after 7 days of osteogenesis induction, *n* = 3. Si‐Cthrc1 −, osteoclast precursors without knockdown of Cthrc1. Si‐Cthrc1 +, osteoclast precursors with knockdown Cthrc1. All data are expressed as the mean ± SD. For A), F), H), I), J), and K), statistical analysis was performed using one‐way ANOVA with Tukey's multiple comparison tests. For C), statistical analysis was performed using two‐tailed unpaired Student's *t*‐tests. ns, no significant difference. ***p < 0.001. (D) was created with BioRender.com.

Osteoclasts not only play a resorptive role in the bone repair process and participate in the cellular degradation of the material but also promote osteogenesis through various coupling factors,^[^ ^37^ ^]^ including bone morphogenetic protein 6, CTHRC1, Ephrin B2, and Platelet‐derived growth factor‐BB.^[^ ^6^, ^38^ ^]^ Dentin or HA has been reported to promote osteogenic differentiation of BMSCs by promoting osteoclast expression of CTHRC1.^[^ ^6b^ ^]^ To verify whether the samples containing HA could effectively promote osteoblast CTHRC1 expression, we seeded osteoclast precursors on PCL, PCH, and PHS. *Cthrc1* expression was detected after 7 days of culture in a complete medium containing M‐CSF (50 ng mL^−1^) and RANKL (30 ng mL^−1^). The qRT‐PCR showed that HA‐containing PCH and PHS significantly promoted *Cthrc1* mRNA expression (**Figure** **3**A).

To verify whether PCH and PHS promote the osteogenic differentiation of BMSCs through CTHRC1, we first transfected a small interfering RNA targeting *Cthrc1* into osteoclast precursors to knock down the expression of *Cthrc1* mRNA (*Si‐Cthrc1*). Immunofluorescence showed that *Si‐Cthrc1* with Cy5 was successfully transfected into the cells (Figure 3B). qRT‐PCR confirmed a significant reduction in *Cthrc1* mRNA expression (Figure 3C). Next, we placed PCL, PCH, or PHS in a trans‐well chamber, seeded the osteoclast precursors in the chambers, and seeded rat BMSCs in the culture dishes under the chambers to construct a coculture model (Figure 3D). After coculture for 7 days, BMSCs were stained with alkaline phosphatase (ALP). The results showed that HA‐containing PCH and PHS significantly promoted the osteogenic differentiation of BMSCs compared with PCL, and the addition of SF‐DFO had no negative impact (Figure 3E), which was confirmed by the absorbance at 560 nm (Figure 3F). The osteogenic differentiation ability of BMSCs induced by PHS was significantly reduced by the knockdown of osteoclast *Cthrc1* mRNA (Figure 3E), which was confirmed by the absorbance at 560 nm (Figure 3F). After 14 days of coculture, alizarin red staining showed that PCH and PHS containing HA significantly promoted calcium salt deposition compared to PCL, and the addition of SF‐DFO had no negative impact (Figure 3G), which was confirmed by the semiquantitative evaluation of alizarin red (Figure 3H). After the knockdown of osteoclast *Cthrc1* mRNA, calcium salt deposition was significantly reduced in the PHS group (Figure 3G), which was confirmed by a semiquantitative evaluation of alizarin red (Figure 3H). After 7 days of coculture, HA‐containing PCH and PHS significantly promoted the expression of osteogenic differentiation‐related genes compared to PCL, including alkaline phosphatase (*Alp*) (Figure 3I), osteocalcin (*Ocn*) (Figure 3J), and collagen type 1 (*Col1*) (Figure 3K). The ability of PHS to induce osteogenesis‐related genes expression was significantly reduced after the knockdown of *Cthrc1* mRNA in osteoclast (Figure 3I–K). In conclusion, these results confirm that HA‐containing PCH and PHS can promote the osteogenic differentiation of BMSCs by promoting osteoclast expression of the coupling factor CTHRC1 and that the addition of SF‐DFO has no negative impact.

### 2.4 PHS Induces Osteogenesis by Promoting Type H Vessel Formation

**Figure 4:**
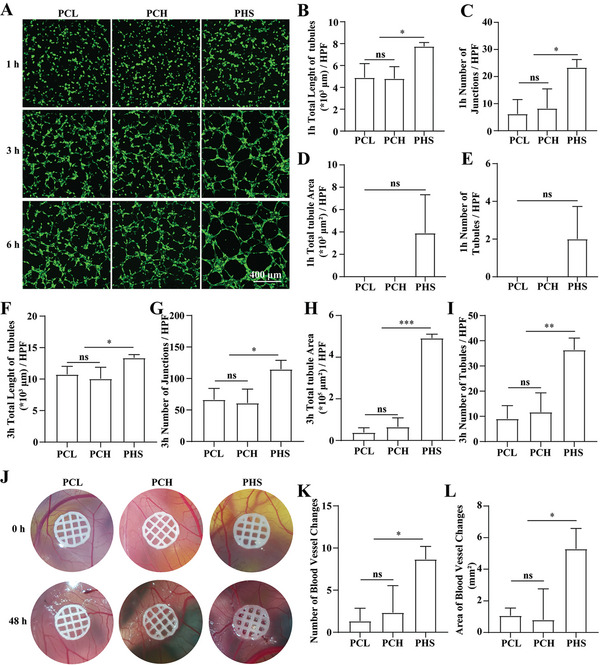
PHS promotes HUVECs tubule formation and CAM angiogenesis. A) Immunofluorescence images of HUVECs after 1, 3, and 6 h of induction using a conditioned medium (supernatant after 1 week of soaking PCL, PCH, or PHS in culture medium). B–E) Statistical analysis of the total length of tubules B), number of junctions C), the total area of tubules D), and number of tubules E) per high‐magnification field at 1 h, *n* = 3. F–I) Statistical analysis of the total length of tubules F), the number of junctions G), the total area of tubules H), and the number of tubules I) per high‐magnification field at 3 h, *n* = 3. J) Images of PCL, PCH, or PHS placed on CAM after 0 and 48 h of incubation. K) Statistical analysis of the change in the number of blood vessels over 48 h, *n* = 3. L) Statistical analysis of the change in the area of blood vessels over 48 h, *n* = 3. All data are expressed as the mean ± SD. For B–I), K), and L), statistical analysis was performed using one‐way ANOVA with Tukey's multiple comparison tests. ns, no significant difference. *p < 0.05, **p < 0.01, ***p < 0.001.

DFO has been shown to promote angiogenesis via a potential mechanism involving the stabilization of HIF‐1*α*, thereby promoting downstream VEGF expression.^[^ ^39^ ^]^ Here, we confirmed that PHS effectively promoted tubule formation in HUVECs and angiogenesis in CAM (**Figure** **4**; and Figure S3, Supporting Information).

**Figure 5:**
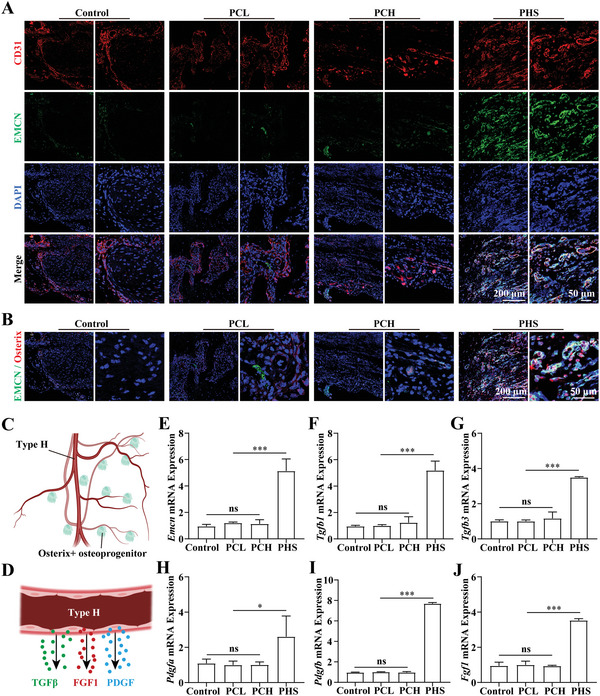
In vivo, PHS promotes callus type H vascular formation and coupling factor expression. A) Immunofluorescence images of type H vessels (EMCN+ CD31+). B) Immunofluorescence images of type H vessels (green) and nearby Osterix+ osteoblasts (red). C) Schematic diagram of type H vessels and nearby Osterix+ osteoprogenitors. D) Schematic diagram of coupling factor secretion by type H vascular endothelial cells. E) Statistical analysis of *Emcn* mRNA expression in calluses, *n* = 6. F–J) Statistical analysis of mRNA expression of coupling factors in calluses, including *Tgfβ1* F), *Tgfβ3* G), *Pdgfa* H), *Pdgfb* I), and *Fgf1* J), *n* = 6. All data are expressed as the mean ± SD. For E–J), statistical analysis was performed using one‐way ANOVA with Tukey's multiple comparison tests. ns, no significant difference. *p < 0.05, **p < 0.01, ***p < 0.001. (C) and (D) were created with BioRender.com

To assess whether PHS can promote type H angiogenesis, we detected the expression of CD31 and Emcn by immunofluorescence staining 4 weeks after PCL, PCH, and PHS implantation into large‐segment bone defects in rats. The control group does not have any implants. The results showed that PHS significantly promoted the formation of type H vessels (**Figure** **5**A), as confirmed by the number (Figure S4A, Supporting Information) and area (Figure S4B, Supporting Information) of CD31+ Emcn+ vessels. The control (no implant), PCL, and PCH groups had little to no Osterix+ bone progenitor cells colonizing the vicinity of the defect (Figure 5B; and Figure S4C, Supporting Information) despite CD31+ angiogenesis (Figure S4B, Supporting Information) due to minimal type H angiogenesis. We examined the mRNA expression at the implantation site four weeks postimplantation. The detection of *Emcn* mRNA expression at the implantation site confirmed that PHS significantly promoted type H angiogenesis (Figure 5E). As mentioned, type H vessels can promote osteogenesis through various coupling factors (Figure 5D). Therefore, we examined the expression of relevant coupling factors at the implantation site. The results showed that the expression of *Tgfβ* (including *Tgfβ1* and *Tgfβ3*) (Figure 5F and G), *Pdgf* (including *Pdgfa* and *Pdgfb*) (Figure 5H,I), and *Fgf1* (Figure 5J) was significantly increased in the PHS group. All of these factors have been shown to effectively promote the regeneration of bone defects.^[^ ^40^ ^]^

The potential mechanism by which DFO promotes type H angiogenesis is the stabilization of HIF‐1*α* (acting on proline hydroxylase and preventing HIF‐1*α* degradation),^[^ ^5^ ^]^ and other investigators and we have reported the potential of DFO to promote bone repair due to the stabilization of HIF‐1*α*.^[^ ^9^, ^41^ ^]^ Here, we demonstrated that PHS stabilized HIF‐1*α* in HUVECs in vitro (Figure S4G, Supporting Information), while promoting downstream *Vegf* expression (Figure S4I, Supporting Information). In vivo, PHS stabilized multiple cellular HIF‐1*α* molecules (Figure S4G, Supporting Information) and promoted downstream *Vegf* expression (Figure S4H, Supporting Information). DFO not only promotes the expression of VEGF in BMSCs but may also directly affect BMSC viability.^[^ ^42^ ^]^ Short‐term administration of DFO may promote BMSC growth (proliferation and colony‐forming capacity) and improve the balance between osteogenic and lipogenic differentiation.^[^ ^42a^ ^]^ However, the direct role of DFO in BMSCs during bone regeneration requires further study. In conclusion, these results confirm that PHS promotes type H angiogenesis and facilitates the expression of multiple osteogenesis‐coupling factors.

### 2.5 PHS Promotes the Regeneration of Large‐Segment Bone Defects in Rats

**Figure 6:**
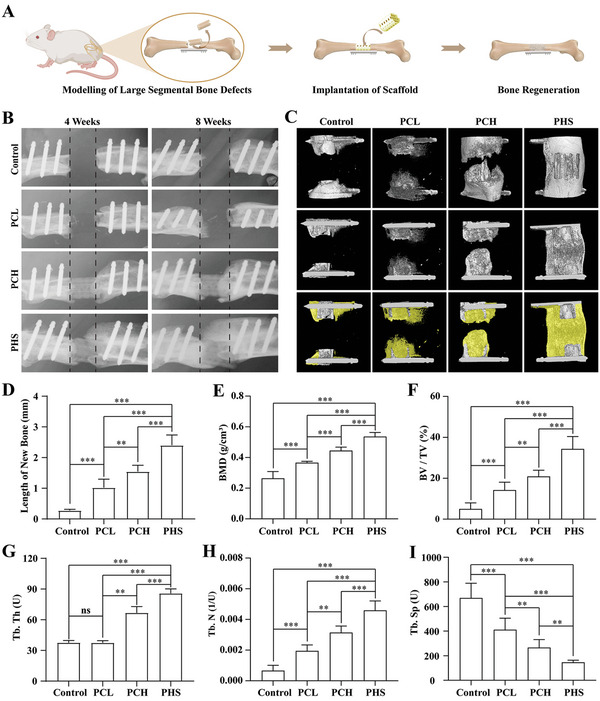
Radiography after PCL, PCH, and PHS implantation into large‐segment bone defects in rats (control, without implants). A) Schematic diagram of the large‐segment bone defect model and scaffold implantation. B) X‐ray images at 4 weeks and 8 weeks after implantation. C) Micro‐CT images at 12 weeks after implantation. D) Statistical analysis of the length of new bone growth between 4 and 8 weeks. Evaluation with X‐ray, *n* = 6. E–I) Statistical analysis of the microstructural parameters of new bone, including BMD, BV/TV, Tb. Th, Tb. N, and Tb. Sp at 12 weeks, *n* = 6. All data are expressed as the mean ± SD. For D–I), statistical analysis was performed using one‐way ANOVA with Tukey's multiple comparison tests. ns, no significant difference. **p < 0.01, ***p < 0.001. (A) was created with BioRender.com.

To assess the ability of PHS to promote the regeneration of large‐segment bone defects, we removed a 6 mm long section from the middle femur of rats and implanted PCL, PCH, and PHS, whereas the control group had no implants (**Figure** **6**A). Bone defect growth was evaluated using X‐rays at 4 and 8 weeks. The results showed that PHS significantly increased the rate of bone regeneration (Figure 6B), which was confirmed by the length of bone growth between 4 and 8 weeks (Figure 6D). By week 12, micro‐CT revealed that the broken ends of the bone defects in the PHS group were largely joined by bone. In the control group, because of the absence of implants, there was almost no new bone formation between the severed ends and only a very small amount of new bone around the internal fixation screws. The PCH group had less new bone than the PHS group but more bone than the PCL group (Figure 6C). Analysis of the microstructural parameters of the regenerated bone using micro‐CT showed that bone mineral density (BMD) (Figure 6E), bone volume/tissue volume (BV/TV) (Figure 6F), and trabecular bone thickness (Tb. Th) (Figure 6G) and trabecular bone number (Tb. N) (Figure 6H) gradually increased, while the trabecular separation gradually decreased (Tb. Sp) (Figure 6I). These results are consistent with the expectation that large‐segment bone defects do not heal without implants.^[^ ^43^ ^]^ Although PCL has no osteo‐inductive or vessel‐inducing properties, it supports bone regeneration and facilitates repair.^[^ ^44^ ^]^ Due to the presence of HA, PCH facilitates osteogenesis by promoting osteoclast formation, resorption, and CTHRC1 expression. PHS greatly facilitates bone regeneration by promoting osteoclast‐coupled and type H vessel‐coupled osteogenesis.

**Figure 7:**
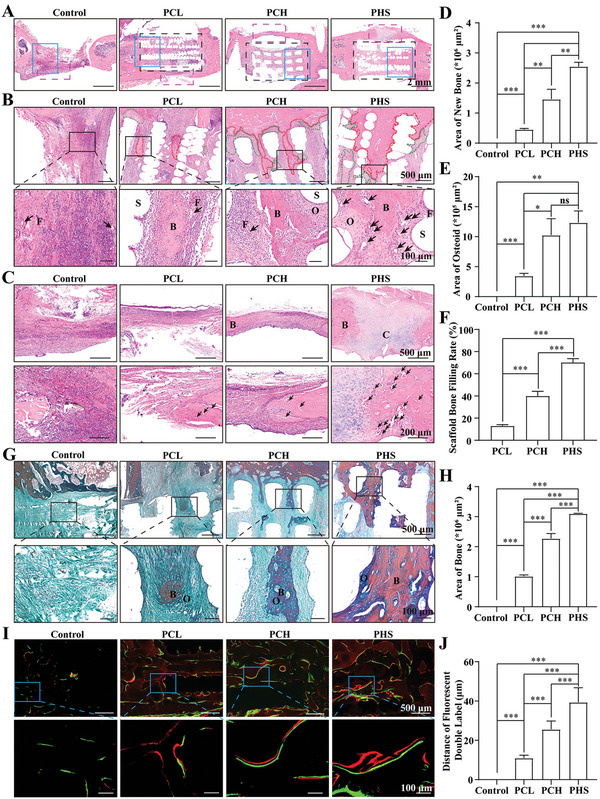
Histological staining. A) Whole image of the large segment of the bone defect. The black dashed box indicates the location of the implanted PCL, PCH, or PHS. B) Enlarged view of the blue box in (A). The red dashed circle shows the newly formed bone growing inside the scaffold, while the green dashed circles show the osteoid. S, scaffold. B, bone. O, osteoid. F, fibrous tissue. Black arrows, blood vessels. C) Enlarged view of the purple dashed box in (A) (external callus). B, bone. C, cartilage. Black arrows, blood vessels. D–E) Statistical analysis of the area of bone D) and osteoid E) growing into the scaffold, *n* = 6. F) Statistical analysis of the filling rate of new bone in the scaffold, *n* = 6. G) Masson staining images. O, osteoid. B, mineralized bone. H) Statistical analysis of the area of mineralized bone growing into the scaffold, *n* = 6. I) Images of calcein‐alizarin red double fluorescein labeling. J) Distance of calcein‐alizarin red double fluorescein labeling, *n* = 6. All data are expressed as the mean ± SD. For D–I), statistical analysis was performed using one‐way ANOVA with Tukey's multiple comparison tests. ns, no significant difference. *p < 0.05, **p < 0.01, ***p < 0.001.

Twelve weeks after scaffold implantation, bone regeneration was assessed by histological staining. Hematoxylin eosin staining (HE) showed that the pores in the PHS group were mainly filled with new bone, the pores in the PCH group were partially filled with new bone, and the pores in the PCL group had only a very small amount of new bone. The control group had almost no new bone (**Figure** **7**A,B), which was confirmed by the area of new bone (Figure 7D), the area of osteoid (Figure 7E), and the scaffold bone filling rate (Figure 7F). The PHS group had significantly more vessels at the bone and osteoid junction than the other groups (Figure 7B, shown by the black arrow). The SF‐DFO released by PHS also significantly promoted bone regeneration around the scaffold (external bone callus), which was entirely connected by new bone and cartilage, whereas the other groups did not show such a connection (Figure 7C). The connection of the outer bone callus helps stabilize the fracture and facilitates the bone repair process.^[^ ^45^ ^]^ At the junction of the cartilage and new bone, the number of vessels was much higher in the PHS group than that in the other groups (Figure 7C, shown by the black arrow). In conclusion, these results confirm that PHS significantly increases the rates of bone regeneration and vascularization.

When osteoblasts begin to form bone, they secrete large amounts of extracellular matrix. As the extracellular matrix increases, osteoblasts are encapsulated by the matrix (osteoid) and promote osteogenesis through the mineralization of the extracellular matrix, such as ALP and matrix vesicles.^[^ ^46^ ^]^ Thus, the mineralization rate of new bone depends on osteoblast function. We evaluated the mineralization of the new bone using Masson staining. The results showed that the newly formed bone was more fully mineralized in the PHS group (Figure 7G), as confirmed by the area of the bone (Figure 7H). At week 4 after scaffold implantation, the rate of calcium salt deposition was measured using the calcein‐alizarin red dual fluorescein labeling method. The results showed that the rate of calcium salt deposition was significantly higher in the PHS group than in the other groups (Figure 7I), as confirmed by the distance between the two fluorescent markers (Figure 7J). In conclusion, these results demonstrate that PHS significantly promoted osteoblast function and increased calcium salt deposition rate, thereby accelerating bone repair.

**Figure 8:**
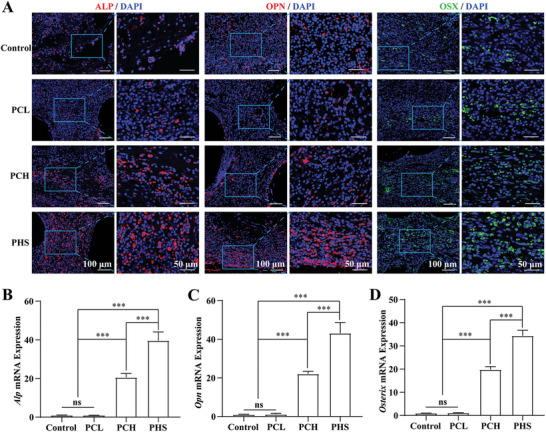
In vivo, PHS promotes the expression of osteogenesis‐related proteins and genes in the callus. A) Immunofluorescence images of ALP, OPN, and OSX expression in calluses. B–D) Statistical analysis of mRNA expression of Alp B), Opn C), and Osx D) in calluses, *n* = 6. All data are expressed as the mean ± SD. For D–I), statistical analysis was performed using one‐way ANOVA with Tukey's multiple comparison tests. ns, no significant difference. ***p < 0.001.

The expression of osteogenesis‐related proteins and genes was examined 4 weeks after PCL, PCH, and PHS implantation in rats with large‐segment bone defects. Immunofluorescence staining showed that PHS significantly promoted the expression of ALP, bone bridge protein (OPN), and Osterix compared with the control (no implant), PCL, and PCH groups (**Figure** **8**A), as confirmed by the number of positive cells per high‐magnification field (Figure S4D–F, Supporting Information). Similarly, the mRNA expression of *Alp*, *Opn*, and *Osterix* was significantly increased at the PCH and PHS implantation sites compared to that in the control and PCL groups, with the greatest increase observed in the PHS group (Figure 8B–D). In conclusion, these results confirm that PHS significantly promotes the expression of osteogenesis‐related genes and proteins and accelerates the bone repair.

### 2.6 PHS Prevents Dislodgement of Internal Fixation Screws

**Figure 9:**
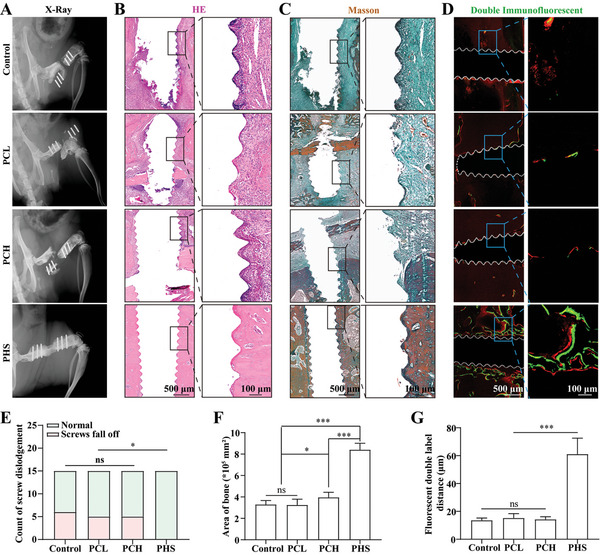
PHS prevents dislodgement of internal fixation screws. A) X‐ray image of screw dislodgement resulting in bone repair failure. The PHS group is normal and well‐repaired. B) HE staining images of the tissue around the screws. The white area indicates the position of the screw. C) Masson staining images of the tissue around the screws. D) Images of calcein‐alizarin red double fluorescein labeling around the screws. E) Statistical analysis of the number of rats in which screw dislodgement occurred in the experiment, *n* = 15. F) Statistical analysis of the bone area around the screws, *n* = 6. G) Statistical analysis of the distance of calcein‐alizarin red double fluorescein labeling around the screw, *n* = 6. (F) and (G) are expressed as the mean ± SD. For (F) and (G), statistical analysis was performed using one‐way ANOVA with Tukey's multiple comparison tests. For (E), statistical analysis was performed using chi‐square test. ns, no significant difference. *p < 0.05, ***p < 0.001.

Aseptic loosening when internal fixation is performed after a fracture is common, especially in elderly patients with osteoporosis.^[^ ^47^ ^]^ The main reason is that the screws do not form a strong connection with the bone.^[^ ^48^ ^]^ Since SF‐DFO can diffuse locally around the internal fixation screw after being released from the scaffold, it is speculated that PHS can promote the binding of the internal fixation screw to the bone tissue. We used a rat osteoporotic large‐segment bone defect model to test this hypothesis. Fifteen female rats were used in each group, and the screws were assessed for dislodgement using radiography 12 weeks after scaffold implantation. The results showed that internal fixation screw dislodgement occurred in control, PCL, and PCH groups, resulting in the failure of bone repair (**Figure** **9**A). However, this was not observed in the PHS group (Figure 9E). Tissue composition around the screws was further evaluated by histological staining at week 12. The PHS was largely surrounded by bone tissue, which formed a strong connection with the screw. In contrast, the remaining groups contained mostly fibrous tissues, that were not firmly attached to the screws (Figure 9B,C). This was confirmed by quantifying the area of bone around the screws (Figure 9F). The calcein‐alizarin red dual fluorescein labeling results confirmed that the rate of calcium salt deposition around the screws was significantly higher in the PHS group than in the other groups (Figure 9D,G). In conclusion, these results show that PHS promotes bone formation around internal screws, thus enhancing their binding to bone tissue.

## 3 Conclusion

We successfully developed a 3D biomimetic calcified cartilaginous callus that induced the formation of type H vessels. It replicates the key raw materials and important links required for normal bone tissue repair in composition and function and has good mechanical properties and biocompatibility. It promotes the long‐term vascularization of healing tissue, osteogenesis‐coupled type H vessels formation, and bone regeneration through various coupling factors. It can promote the formation and differentiation of osteoclasts and can be resorbed by osteoclasts, creating a suitable repair environment for osteoblast osteogenesis and promoting the osteogenic differentiation of BMSCs through coupling factors. It significantly promotes bone callus vascularization and regeneration after implantation into large‐segment bone defects in rats. In addition, it enhances the binding of the internal fixation screws to the bone and prevents the dislodgement of internal fixation screws during bone repair, which is important for clinical bone repair, particularly in elderly patients with osteoporosis. In conclusion, a 3D biomimetic calcified cartilaginous callus inspired by the biological process of normal bone repair was developed to effectively promote the regeneration of large‐segment bone defects and has great clinical translational value for bone tissue engineering and regenerative medicine.

## 4 Experimental Section

### Synthesis of HA‐SF‐DFO

The synthesis method for SF‐DFO has been described in the previous studies.^[^ ^14^ ^]^ The molecular weight of SF‐DFO was 775.84. The HA used in this study was a synthetic nanocrystalline with a particle size of less than 200 nm (BET) and a molecular weight of 502.31 (677 418, Sigma). For the synthesis of HA‐SF‐DFO, SF‐DFO was first dissolved in dimethyl sulfoxide (DMSO) (276 855, Sigma) to make a final concentration of 2.5 mg mL^−1^, and then 50 mg of HA nanopowder was added to each milliliter of the solution. Centrifuge at 12 000 g for 5 min after 30 min of continuous stirring at room temperature. After removal of the supernatant, the powder was washed three times with DMSO to remove unbound SF‐DFO. The residual DMSO was removed by freeze‐drying to obtain HA‐SF‐DFO powder.

### Study on the Efficacy of SF‐DFO Binding with HA

A series of SF‐DFO concentrations (10, 5, 2.5, 1.25, 0.625, and 0 mg mL^−1^) were used to investigate the effect of different concentrations of the drug on binding efficacy. The HA nanopowder (50 mg of HA nanopowder was added to each milliliter of the solution. After 30 min of continuous stirring at room temperature, the mixture was centrifuged at 12 000 g for 5 min. The mass of HA‐bound SF‐DFO was determined by measuring the remaining SF‐DFO concentration in the supernatant. The ability of DFO to change the color of the ferric chloride solution was used to detect the concentration of SF‐DFO. The assay solution was prepared by dissolving ferric chloride hexahydrate in DMSO to a final 10 mg mL^−1^ concentration. 50 µL of the prepared assay solution was first added to a 96‐well cell culture dish, followed by 50 µL of the configured SF‐DFO solution of different concentrations. After thorough mixing, absorbance was measured at 560 nm using a filtered multifunctional enzyme marker (Infinite F200 Pro, Switzerland). The measured absorbance and known SF‐DFO concentrations were used to plot a standard curve. The absorbance of the solution was compared with a standard curve to calculate the SF‐DFO concentration. After determining the most suitable SF‐DFO concentration, a series of time gradients (0.5, 1, 2, 4, 8, and 12 h) were used to explore the effect of time duration on binding. The detection method used was the same as described above. In the above tests, three replicates of each test were performed.

### Fabrication of 3D Biomimetic Calcified Cartilaginous Callus

The molecular weight of PCL used in this study was 80 000 (440 744, Sigma). HA‐SF‐DFO powder was thoroughly mixed with PCL powder to prepare cylindrical PCL/HA‐SF‐DFO 3D biomimetic calcified cartilaginous callus (PHS) with a diameter of 4 mm and height of 6 mm using a pneumatic melting device. The scaffolds were fabricated using an in‐house pneumatic fused‐deposition system mounted to an additive manufacturing device with a nozzle diameter of 200 µm. The printed filament diameter was set to 200 µm and the filament spacing to 400 µm. The liquefier temperature was set to 90 °C. The mass percentages of HA and PCL used in the PCH scaffolds were 20% and 80%, respectively. The mass percentages of HA‐SF‐DFO and PCL used in the PHS scaffolds were 20% and 80%, respectively. A cell lattice structure with a 0/90° lamination pattern and continuous contour filaments was used to fabricate a 3D porous spatial structure that mimicked the calcified cartilaginous callus.

### Characterization of HA‐SF‐DFO Microparticles and 3D Biomimetic Calcified Cartilaginous Callus

Dynamic scattering light (DLS, Malvern Instruments Ltd, UK) was utilized to determine the size distribution of the HA and HA‐SF‐DFO microparticles. A Fourier‐transform infrared spectrometer (Agilent Cary630, Malaysia) was used to obtain the infrared spectra of HA, SF‐DFO, and HA‐SF‐DFO. In order to visualize the 3D structure of the PHS, the PHS scaffold was scanned using a high‐resolution micro‐CT (Scanco Medical, Switzerland) at 5 µm isometric resolution (70 kV and 130 µA radiation source with 0.5 mm aluminum filter) and then reconstructed in 3D using Imaris software. The macropores and microstructure of the pore walls were characterized using scanning electron microscopy (SEM, QUANTA 250, FEI, US). XPS was used to analyze SF‐DFO in PHS. EDS analyzed the scaffold sections’ elemental distribution and composition using scanning electron microscopy (SEM‐EDS, QUANTA 250, FEI, US). The PCL, PCH, and PHS scaffolds were printed into cylindrical shapes with a diameter of 4 mm and a height of 15 mm for compression testing. Compression measurements of the scaffolds were performed along the print axis (*z*‐axis) using a universal testing machine (Shimadzu, AG‐2000A, Japan) with displacement control (4 mm min^−1^) under ambient conditions. In the above tests, three replicates of each test were performed.

### Scaffold Degradation and Drug Release

The scaffold was immersed in 0.01 m PBS and then shaken at 37 °C on a shaker at 60 HZ to assess its degradation properties. The scaffolds were removed at fixed times and weighed after vacuum drying. In order to evaluate drug release, the PHS scaffolds were immersed in 10 mL 0.01 m PBS solution of pH 7.4 at 37 °C and then shaken at 60 HZ. After integration with a ferric chloride solution (FeCl_3_, 10 mg mL^−1^), the amount of released SF‐DFO was determined by measuring the absorbance at 560 nm using a filter‐type multifunctional enzyme labeling instrument (Infinite F200 Pro, Switzerland), according to the standard SF‐DFO calibration curve. A FeCl_3_ solution (10 mg mL^−1^) was mixed with the HA‐SF‐DFO microparticles and shaken well to measure the total amount of SF‐DFO in the HA‐SF‐DFO microparticles. The absorbance of the supernatant was measured after centrifugation. For measuring the SF‐DFO released from HA‐SF‐DFO microparticles, HA‐SF‐DFO microparticles were dispersed in PBS and shaken for 5 min at 37 °C. The supernatant was collected by centrifugation at 12 000 g for 5 min at a fixed time, and the SF‐DFO content was determined using the method mentioned above. In the above tests, three replicates of each test were performed.

### In Vitro Cell Biocompatibility Measurements

Rat BMSCs were used to study cell adhesion and proliferation on the scaffolds. In brief, after the euthanasia of 8‐week‐old rats, the femoral and tibial bone marrow was flushed out and dispersed with Minimum Essential Medium *α* (MEM *α*, 12 571 048, Gibco) containing 10% fetal bovine serum and 1% penicillin‐streptomycin. After 24 h of incubation, the upper layer of unadhered cells was removed, and the culture was continued for 5 days to obtain primary BMSCs. To purify BMSCs, the culture was continued for 3 generations. The cells were cultured in MEM *α* with 10% fetal bovine serum (FBS, 10 099 141, Gibco) and 1% penicillin‐streptomycin (PS, 15 140 148, Gibco) in an incubator with 5% CO_2_ at 37 °C. The scaffolds were first soaked in 75% alcohol for 30 min and then in MEM *α* containing 10% fetal bovine serum for 10 min. Cells were seeded at a density of 5 × 10^4^ cells mL^−1^ in a 24‐well tissue culture plate and grown on the surface of the scaffolds. A Live/Dead Cell kit (L3224, Invitrogen) was used to analyze scaffold viability. After 1, 3, and 7 days of cultivation, Cells attached to the scaffolds were stained with 500 µL of combined dyes in an incubator for 20 min and then examined under a fluorescent microscope (ZEISS, Axio, Germany). Similarly, cytotoxicity and cell proliferation were analyzed using the CCK‐8 assay at designated time points (1, 3, and 7 days). 0.5 mL of fresh MEM *α* containing a 10% CCK‐8 solution (C0037, Beyotime) was added to each well. After 2 h, 100 µL of the mixed medium was transferred to a 96‐well plate. The absorbance of the solution was measured at 450 nm using an enzyme marker (Infinite F50, TECAN, Switzerland). To assay the cytotoxicity of SF‐DFO, BMSCs were seeded in 96‐well culture plates. The cells were then cultured in media containing different concentrations of SF‐DFO and assayed using the CCK8 method described above. At the designated time points (1, 3, and 7 days), cells were washed three times with 0.01 m PBS (pH 7.4) and fixed with 4% paraformaldehyde solution (P0099, Beyotime) for 10 min at room temperature. The cells were permeabilized with 0.2% v/v Triton X‐100 (P0096; Beyotime) for 15 min and washed with PBS. Afterward, cells were treated with 0.5 mL of 5 µg mL^−1^ Alexa Fluor 594 phalloidin (A12381, Invitrogen) and 0.5 mL of 10 µg mL^−1^ 4, 6‐Diamidino‐2‐phenyindole dilactate (DAPI, D3571, Invitrogen) for 5 min at room temperature, respectively. Finally, the labeled cells on the scaffold were observed using a laser‐scanning confocal microscope (LSCM, LSM800, ZEISS, Germany). High‐resolution z‐stack imaging was performed. In the above tests, three replicates of each test were performed.

### Osteoclast Induction and Resorption Assay

Bone marrow‐derived mononuclear cells (BMM) were used to induce osteoclast formation and resorption assays. In brief, after the euthanasia of 8‐week‐old rats, the femoral and tibial bone marrow was flushed out and dispersed with MEM *α* (12 571 048, Gibco). After 24 h of incubation, the upper layer of unadhered cells was collected, and the BMM was adhered to using 50 ng mL^−1^ M‐CSF (ab269198, Abcam). The next day, the upper layer of unadhered cells was removed, and the culture was continued for 48 h. Cells were scraped off using a cell scraper to induce osteoclast formation. For in vitro osteoclast formation and resorption, PCL, PCH, and PHS were melted into thin sheets. After placing the sheet in a 24‐well culture plate, the BMM was grown on the sheet at a density of 5 × 10^4^ cells mL^−1^. MEM *α* containing 50 ng mL^−1^ M‐CSF and 30 ng mL^−1^ RANKL (ab69517, Abcam) was used for 16 days of induction. After fixation with 4% paraformaldehyde, TRAP staining was performed to observe the osteoclasts. To detect osteoclast‐associated gene expression, TRIzol reagent was used to extract mRNA and perform qRT‐PCR. The primer sequences are listed in Table S1 (Supporting Information). In the above tests, three replicates of each test were performed.

### TRAP Staining

Trap staining was performed according to the manufacturer's protocol (387, Invitrogen). 0.5 mL Fast Garnet GBC Base Solution (7.0 mg mL^−1^, in 0.4 mol L^−1^ hydrochloric acid with stabilizer, 3872, Invitrogen) and 0.5 mL sodium nitrite solution (0.1 mol L^−1^, 914, Invitrogen) are mixed by gentle inversion for 30 s and then stand 2 min. TRAP dye solution consists of the following components: 45 mL deionized water prewarmed to 37 °C, 1.0 mL of the previously prepared mixture, 0.5 mL Naphthol AS‐Bl Phosphate Solution (12.5 mg mL^−1^, 3871, Invitrogen), 1.0 mL^−1^ (+)‐Tartrate buffer (0.335 mol L^−1^, pH 4.9 ± 0.1, 3873, Invitrogen), and 2.0 mL^−1^ Acetate buffer (2.5 mol L^−1^, pH 5.2 ± 0.1, 3863, Invitrogen). The samples were fixed for 30 s using 4% paraformaldehyde. Wash three times with deionized water. Incubate for 1 h at 37 °C, protected from light, using the previously configured TRAP staining solution. After 1 h, the slides were rinsed thoroughly in deionized water and counterstained for 2 min in Hematoxylin Solution (GHS3, Invitrogen). The cells were rinsed in alkaline tap water for several minutes to remove the blue nuclei. Air‐drying and microscopic evaluation After staining, the cells were observed and photographed using a microscope (Zeiss, Germany) and statistically analyzed using ImageJ. Three samples of each material were analyzed.

### SEM of Osteoclasts and their Resorption Lacunae

The SEM method for osteoclasts and their resorption lacunae was performed according to the protocol of Schumacher et al.^[^ ^48^ ^]^ Briefly, to observe resorption lacunae, samples were soaked in cell lysis buffer (1% Triton X‐100 in PBS) for 50 min and then sonicated (40 KHz, 5 min) to remove cells from the unfixed samples. Cells were fixed using a 25% aqueous solution of 1,5‐glutaraldehyde (G5882; Sigma). Both the samples were dehydrated by immersion in a series of ethanol solutions and subjected to critical‐point drying (EM CPD300, Germany). The surface morphologies of the samples were observed using scanning electron microscopy (SEM, Sirion 200, USA). The areas of the ten absorption lacunae for each sample were quantified using the ImageJ software, and three samples of each material were quantified. The fissure depths were determined using a profilometer (Alpha‐Step D‐600; USA). Ten absorption lacunae were measured for each sample, and three samples of each material were measured.

### Small Interfering RNA (Si‐RNA) Transfection and Osteoclast‐BMSCs Coculture

Osteoclasts and BMSCs were extracted and cultured as previously described. In order to transfect Si‐Cthrc1 in osteoclast precursors, Si‐Cthrc1 was transfected using Entranster‐R4000 reagent (4000‐4, Engreen Biosystem, China). The sequence of Si‐Cthrc1 is listed in Table S1 (Supporting Information). Sequences labeled with cy5 were used as transfection controls. Briefly, siRNA and Entranster‐R4000 reagents were mixed separately by dilution with serum‐free diluent. The mixture was then added to a complete medium (containing 10% FBS) and transfected. Forty‐eight hours later, transfection was verified by immunofluorescence and qRT‐PCR. The qRT‐PCR primers used to amplify Cthrc1 are listed in Table S1 (Supporting Information). To study the effect of osteoclast CTHRC1 on BMSCs, osteoclast‐BMSCs were cocultured in a trans‐well chamber (CLS3396, Corning). Thin sheets of PCL, PCH, and PHS were placed in the chamber, osteoclast precursors were planted in the chamber, and BMSCs were seeded in culture dishes (Figure 3D). A complete culture medium containing 50 ng mL^−1^ M‐CSF and 30 ng mL^−1^ RANKL was used. A complete culture medium containing 50 µg mL^−1^ ascorbic acid, 10 mm *β*‐glycerol phosphate, and 10^−8^ m dexamethasone was used in the petri dish. After 7 days of coculture, total RNA was collected for qRT‐PCR to detect the expression of osteogenesis‐related genes. The primers used are listed in Table S1 (Supporting Information). In the above tests, three replicates of each test were performed.

### Alkaline Phosphatase Staining and Alizarin Red s Staining

Alkaline phosphatase (ALP) staining was performed on BMSCs after 7 days of coculture. The cells were fixed with a 4% paraformaldehyde solution and washed three times with PBS. Wash the fixed cells with PBS three times, and then use the alkaline phosphatase detection kit (P0321S, Beyotime) for ALP staining. After incubating for 20 min at room temperature, the cells were washed with distilled water. The samples were air‐dried, and images were acquired using a digital camera. To quantify ALP staining, absorbance was measured at 560 nm. Alizarin red staining was performed on the BMSCs after 14 days of coculture. The cells were fixed with a 4% paraformaldehyde solution and washed three times with PBS. The fixed cells were washed with distilled water to remove salt residues. A solution of 2% w/v Alizarin Red S (ARS) with a pH adjusted to 4.2 was added to cover the entire surface of the wells. After incubation for 10 min at room temperature, the excess ARS was washed off with distilled water. The samples were air‐dried, and images were acquired using a digital camera. Extraction was performed to quantify the orange‐red color of alizarin red by adding a 70% ethanol solution of 10 mm HCl to the stained Petri dishes and incubating for 30 min. The absorbance of the extracts was measured at 450 nm. In the above tests, three replicates of each test were performed.

### HUVECs Tubule Formation Assay

To assess the ability of the PHS scaffold promotes angiogenesis, growth factor‐reducing Matrigel (100 µL per well) (354 230, Corning) was used to induce tube formation on 24‐well tissue culture plates. Briefly, the frozen Matrigel was melted on ice, then spread evenly in a 24‐well cell culture plate, and incubated for 30 min in an incubator (5% CO_2_ and 37 °C).^[^ ^49^ ^]^ Then HUVECs expressing green fluorescent protein were implanted in each well. Scaffolds were soaked in fresh DMEM (11 965 092, Gibco) for seven days by shaking at 4 °C in a refrigerator, and the extracted supernatant was used as a medium for HUVECs. The cells were then incubated at 5% CO_2_ in a 37 °C incubator and dynamically observed, and pictures were obtained using laser scanning confocal microscopy (LSCM, LSM800, ZEISS, Germany) at the 1st, 3rd, and 6th h, respectively. The tubule formation tube data were statistically analyzed with the ImageJ plugin Angiogenesis Analyzer [The relevant plugin can be downloaded from https://imagej.nih.gov/ij/macros/toolsets/]. In the above tests, three replicates of each test were performed.

### Chick Embryo Chorionic Villus Allantoic Membrane Angiogenesis Assay

Fertilized eggs were incubated in incubators at 37–39 °C and 60–70% relative humidity for 10 days, and then the air chambers were searched for and marked in a dark room using a flashlight. The outer membrane was moistened with saline, and the outer layer of the allantoic membrane was carefully removed with forceps, not damaging the allantoic membrane. The scaffold was placed on the part of the egg with relatively few blood vessels, the air chamber was closed with a sterile dressing, and the chicken embryo was incubated for 2 days to observe the growth of blood vessels. Vascular morphology was observed by photography with a Canon camera (Mark IV, Japan), and images were processed using ImageJ software. In the above tests, three replicates of each test were performed.

### qRT‐PCR

Total RNAs from MSCs, HUVECs, osteoclasts, and tissues were extracted using TRIzol Reagent (15 596 026, Ambion). Relative RNA expression levels were evaluated using qRT‐PCR, and the housekeeping gene *Actin* was used as a loading control. All PCR amplifications were performed in a final reaction mixture (20.0 µL), and the relative primer sequences are listed in Table S1, Supporting Information. The amplification reaction was performed using TB Green Premix Ex Taq (RR420A, Takara) for 40 cycles, and relative expression was calculated according to the 2^−ΔΔCt^ method.

### Experimental Animal and Large‐Segment Bone Defects

The experimental procedures, housing, and animal care were approved and performed following the animal experimentation regulations of the Animal Ethics Committee of Shanghai Jiaotong University (SYXK (Hu) 2018‐0027). Eight‐week‐old male and female Sprague Dawley (SD) rats were purchased from Beijing Weitong Lihua Laboratory Animal Technology Co., Ltd. Shanghai Branch (Shanghai, China) and housed under standard pathogen‐free animal facilities in a temperature‐controlled room (24 ± 2 °C). After 2 weeks of adaptation, 300–350 g SD male rats were used to establish a large segmental bone defect model of weight‐bearing bones. Rats were anesthetized with 2.5% sodium pentobarbital (40 mg kg^−1^ body weight). After sterilization, a sterile towel was laid down, and the skin and muscle layers were incised from the thigh to expose the femur. The internal fixation plate was fixed to the femur using screws, and the wire saw was used to remove the middle section of the bone, which was ≈6 mm in length. The PCL, PCH, and PHS scaffolds were then implanted separately. The control group did not have implants, and muscle and skin sutures were performed. The animals were assessed for bone growth by radiography (Delmedical, USA) at 4 and 8 weeks postoperatively. The length of bone growth between 4 and 8 weeks was measured using the ImageJ software after using X‐rays. Six rats were assigned to each group. At week 4, six rats per group were used to assess the rate of calcium salt deposition by the calcein‐alizarin red dual fluorescein labeling method (described in detail later). Six rats per group were euthanized using CO_2,_ and the femur was removed and fixed for immunostaining (described in detail later). Six rats per group were euthanized using CO_2,_ and the scaffolds were removed for RNA extraction for qPCR (as described previously). At week 12 postoperatively, the animals were euthanized with CO_2,_ and the femur was removed for micro‐CT (Scanco Medical, Switzerland) evaluation after fixation using 4% paraformaldehyde. The accuracy of the scan is set to a continuous layer sweep of 20 µm (70 kV and 130 µA radiation source with a 0.5 mm aluminum filter). Scanco software was used to analyze the bone microstructure. BMD, BV/TV, Tb.N, and Tb.Sp were determined. Female rats were anesthetized and sterilized for osteoporotic large‐segment bone defects as described above. After 2 weeks of acclimatization, the dorsal skin was cut, and the muscle layer was tonically separated. The ovaries were excised and ligated. The muscles and skin were sutured, and feeding was continued. Two months later, the PCL, PCH, and PHS scaffolds were implanted as previously described. The control group did not have implants. Fifteen rats were used in each group, and the number of animals with loose screws was counted using radiographs obtained 12 weeks after scaffold implantation. The animals were euthanized using CO_2,_ and the femurs were removed and fixed in 4% paraformaldehyde.

### Histological and Immunohistochemical Analysis

For bone histological analysis, femurs were fixed in 4% paraformaldehyde for 48 h, followed by decalcification in 10% EDTA for 8 weeks, then embedded in paraffin, and 5 µm thick sections were used for HE staining, Masson staining, and immunohistochemical analysis. For immunostaining, bone sections were dried, permeabilized in 0.3% Triton X‐100 for 10 min, blocked in 5% goat serum for 30 min at room temperature, and probed with primary antibodies diluted in 5% goat serum in PBS overnight at 4 °C. The following primary antibodies were used: HIF1‐*α* (NB100‐105, Novus, 1:200), CD31 (NB100‐2284, Novus, 1:200), EMCN (PA5‐115178, Invitrogen, 1:200), Alkaline phosphatase (NB110‐3638, Novus, 1:200), Osteopontin (ab63856, Abcam, 1:200), Osterix (sc‐393060, Santa Cruz Biotechnology, 1:200). After primary antibody incubation, the sections were washed with PBS and incubated with the appropriate Alexa Fluor‐coupled secondary antibodies for 1 h at room temperature. The cell nuclei were stained with DAPI (D9542, Sigma). Finally, the sections were analyzed using LSCM. The signal intensity was quantified using the ImageJ software. Six sections from different rats were analyzed in each group. For Masson staining, the prepared thin sections were dewaxed and stained with Weigert iron hematoxylin staining solution (HT1079, Sigma) for 5–10 min. Cells were differentiated in an acidic ethanol differentiation solution (1% hydrochloric acid in alcohol, Sigma) for 5–15 s and washed with water. The samples were then turned blue in a 1% lithium carbonate aqueous solution (G1841, Solarbio) for 3–5 min and washed with distilled water for 1 min. Stain with Ponceau acid fuchsin staining buffer (LZ‐12686, Guidechem) for 5–10 min, and wash with a weak acid working solution (0.1–0.3% acetic acid solution, Sigma) for 1 min. Washing with a phosphomolybdic acid solution (20 wt% in ethanol, 319 279, Sigma) for 1–2 min, wash with a weak acid working solution for 1 min, and put it in aniline blue staining solution (2.5% in 2% acetic acid, B8563, Sigma) for 1–2 min. Wash with a weakly acidic working solution for 1 min. After dehydration, the sheets were sealed with a neutral resin. HE and Masson staining images were acquired using a high‐throughput scanner (Aperio AT2, Leica), and the bone area was calculated using Image J software. Six sections from different rats were analyzed in each group.

### Calcein‐Alizarin Red Dual Fluorescein Labeling

Double fluorescein labeling was performed 4 weeks after scaffold implantation into large segmental bone defects. Calcein (30 mg kg^−1^) was injected 10 days before euthanasia, and alizarin red (30 mg kg^−1^) was injected 3 days before euthanasia. Samples were fixed in 70% ethanol for 48 h, embedded, and hard tissues were sectioned using the EXAKT system (Germany). The distance between the double fluorescein markers was calculated using ImageJ software after photographing using LSCM. Six sections from different rats were analyzed in each group, with fluorescence spacing measured at ten sites per section.

### Statistical Analysis

All studies were evaluated in at least three independent experiments for each condition to ensure reproducibility. Data are expressed as mean ± SD. Significant differences between different groups were determined using two‐tailed unpaired Student's *t*‐tests for two‐group comparisons and one‐ or two‐way analysis of variance (ANOVA) with post hoc Tukey's test for multiple‐group comparisons. For all statistical tests, significance was defined as *p* ≤ 0.05. Statistical analyses were performed using GraphPad Prism version software.

## Conflict of Interest

The authors declare no conflict of interest.

## Supporting information

Supporting Information

Supplemental Video 1

## Data Availability

The data that support the findings of this study are available from the corresponding author upon reasonable request.
