# Supplementary material for: 3D Biomimetic Calcified Cartilaginous Callus that Induces Type H Vessels Formation and Osteoclastogenesis
Source: Adv Sci (Weinh). 2023 Mar 31;10(16):2207089. doi: 10.1002/advs.202207089 (PMC10238192; doi:10.1002/advs.202207089)
Supplement: Supplementary file 1 — Supporting Information [file ADVS-10-2207089-s001.pdf]

ADVANCED SCIENCE
Open Access

Supporting Information

for *Adv. Sci.*, DOI 10.1002/advs.202207089

3D Biomimetic Calcified Cartilaginous Callus that Induces Type H Vessels Formation and Osteoclastogenesis

Minglong Qiu, Changwei Li, Zhengwei Cai, Cuidi Li, Kai Yang, Nijiati Tulufu, Bo Chen, Liang Cheng, Chengyu Zhuang, Zhihong Liu, Jin Qi, Wenguo Cui* and Lianfu Deng**

3D Biomimetic Calcified Cartilaginous Callus that Induces Type H Vessels Formation and Osteoclastogenesis

Minglong Qiu[#], Changwei Li[#], Zhengwei Cai[#], Cuidi Li, Kai Yang, Nijiati Tulufu, Bo Chen, Liang Cheng, Chengyu Zhuang, Zhihong Liu, Jin Qi, Wenguo Cui*, Lianfu Deng**

Department of Orthopaedics, Shanghai Key Laboratory for Prevention and Treatment of Bone and Joint Diseases, Shanghai Institute of Traumatology and Orthopaedics, Ruijin Hospital, Shanghai Jiao Tong University School of Medicine, 197 Ruijin 2nd Road, Shanghai 200025, P. R. China.

[#] These authors contributed equally to this work

*Corresponding authors:

Email addresses: jinjin838@hotmail.com (J. Qi), wgcui80@hotmail.com, (W. Cui), lf_deng@126.com (L. Deng)

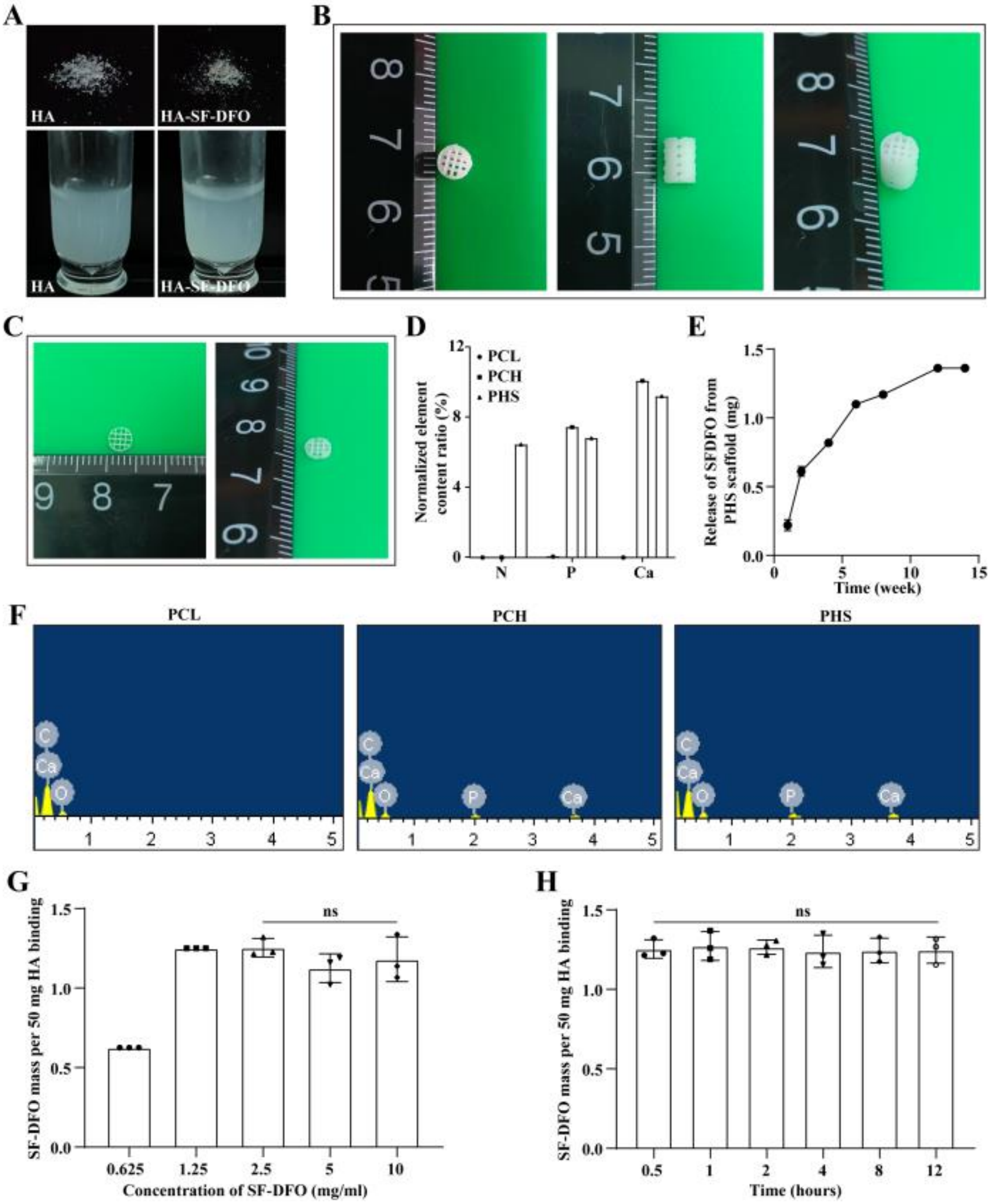

Figure S1. Characterization of PHS. A) Images of HA and HA-SF-SFO powders and their dispersion in water. B) Photograph of the 3D scaffold used to repair the large segmental bone defect. C) Photograph of the 3D scaffold used for cell culture. D) Percentages of the normalized elemental content of N, P, and Ca within each group of scaffolds. E) Mass curve of SF-DFO release from PHS with a time delay, $n = 3$. F) SEM–EDS showing the elemental composition inside the scaffold. G) Statistical graph of the mass of SF-DFO that can be bound by 50 mg of HA in different concentrations of SF-DFO solution, $n = 3$. H) Statistical plot of the mass of SF-DFO

that can be bound by HA in combination with SF-DFO at different times, $n = 3$. All data are expressed as the mean ± SD. For G) and H), statistical analysis was performed using one-way ANOVA with Tukey's multiple comparison tests. ns, no significant difference.

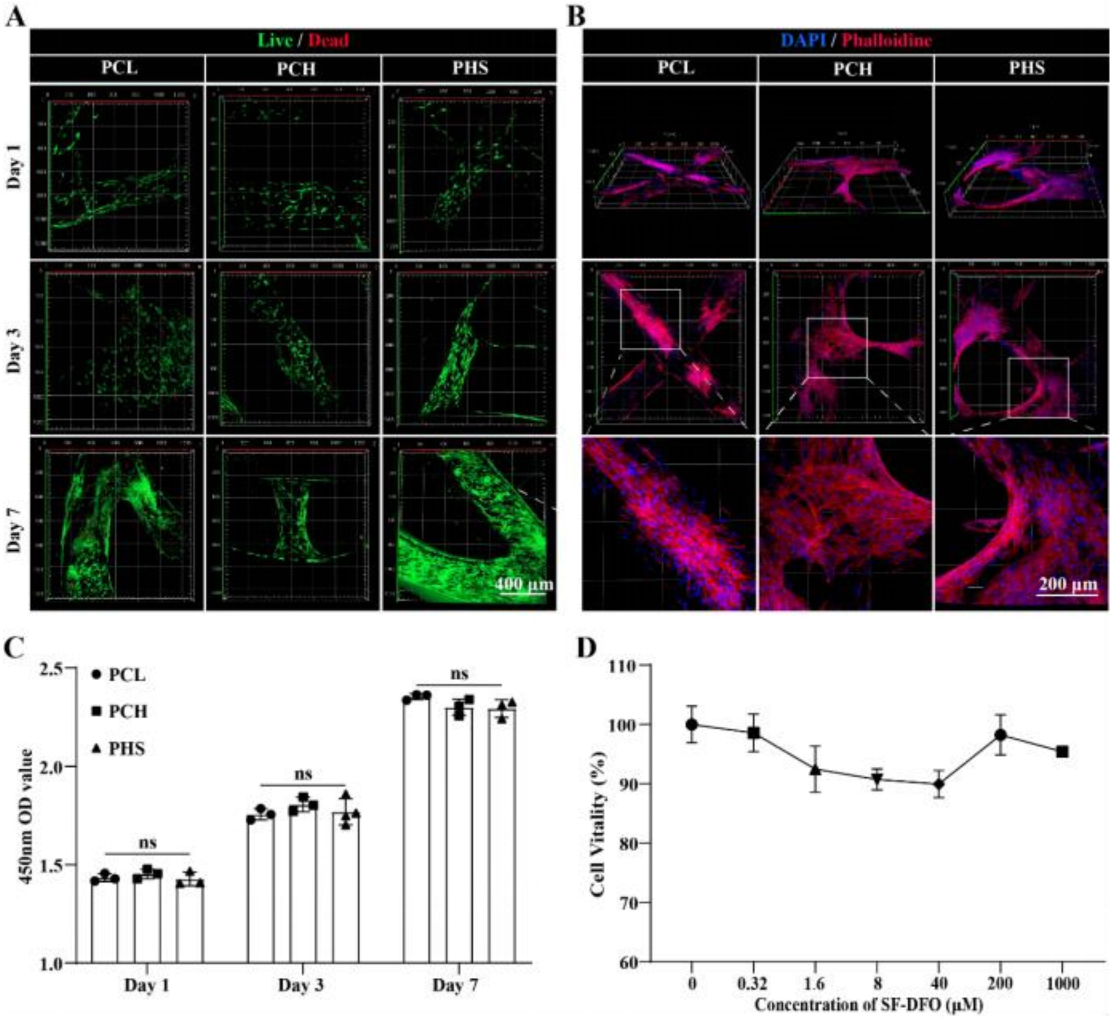

Figure S2. Biocompatibility of PCL, PCH, and PHS. A) Live/dead staining images of rat bone marrow stromal stem cells (BMSCs) grown on PCL, PCH, and PHS 1, 3, and 7 days after implantation. B) Cytoskeletal staining images of BMSCs on PCL, PCH, and PHS. C) Cell Counting Kit-8 (CCK-8) to detect the proliferation rate of cells after 1, 3, and 7 days of BMSC growth on PCL, PCH, and PHS, $n = 3$. D) Curves of the effects of different concentrations of SF-DFO on the viability of BMSCs, $n = 3$. All data are expressed as the mean ± SD. For C, statistical analysis was performed using one-way ANOVA with Tukey's multiple comparison tests. ns, no significant difference.

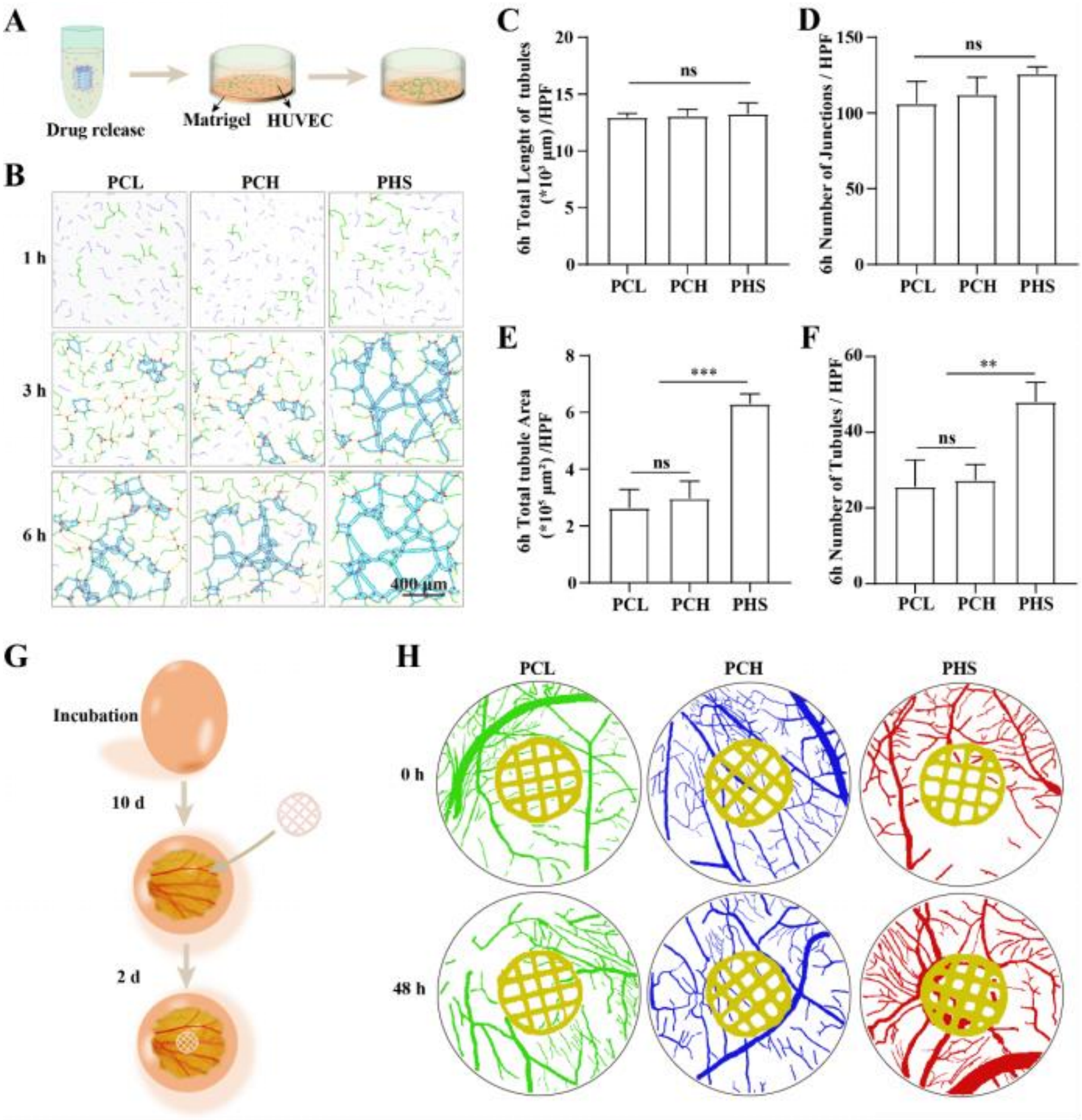

Figure S3. PHS promotes human umbilical vein endothelial cell (HUVEC) tubule formation and chick embryo chorioallantoic membrane (CAM) angiogenesis. A) Scheme of drug release and promotion of human umbilical vein endothelial cell (HUVEC) tubule formation. B) Images of tubule formation were analyzed and calculations were performed with ImageJ. C - D) Statistical analysis of the total length of tubules (C), the number of junctions (D), the total area of tubules (E), and the number of tubules (F) per high-magnification field at 6 hours, $n = 3$. G) Scheme of the chick embryo CAM angiogenesis assay. H) Depiction of CAM vessels. All data are expressed as the mean ± SD. For C – F), statistical analysis was performed using one-way ANOVA with Tukey's multiple comparison tests. ns, no significant difference.

**$p < 0.01$, ***$p < 0.001$.

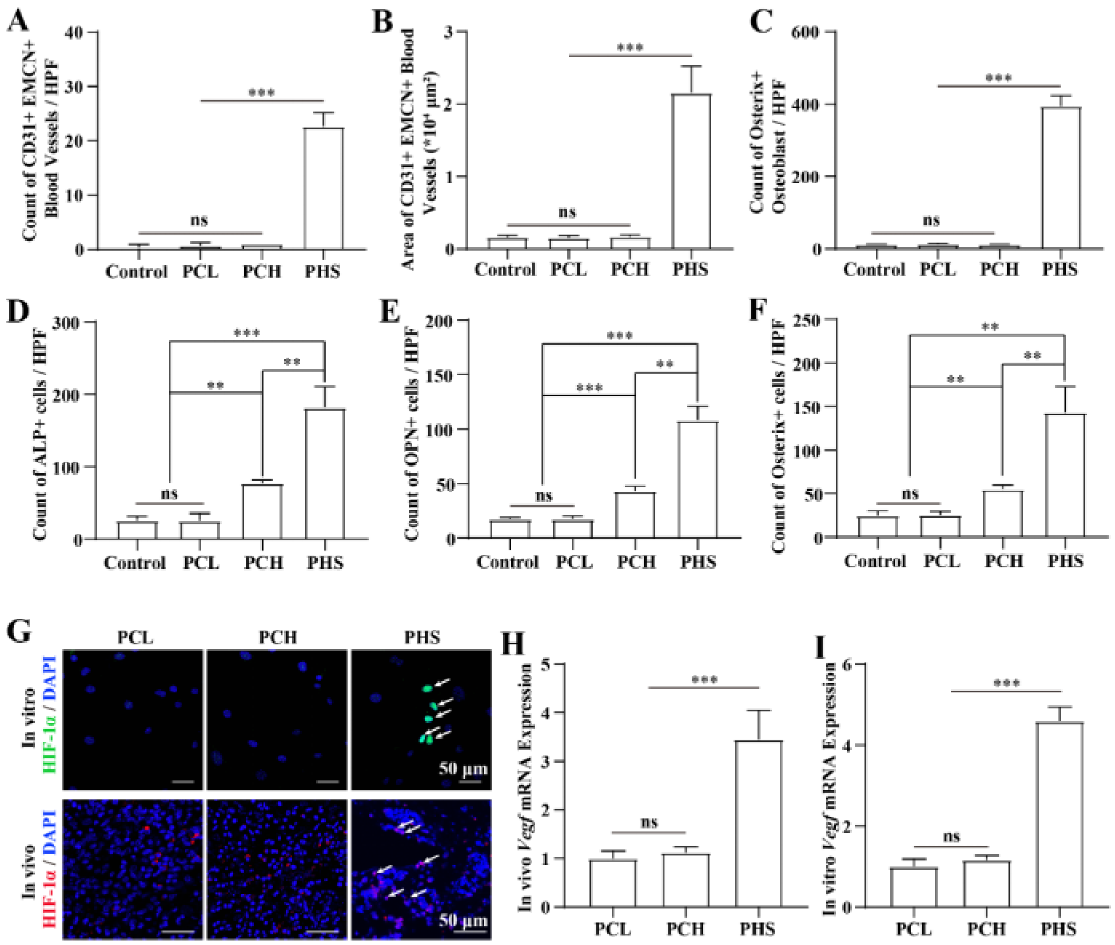

Figure S4. PHS promotes type H angiogenesis and osteogenesis coupling. A) Count of CD31+ and EMCN+ vessels in Figure 3, $n = 6$. B) Areas of CD31+ and EMCN+ vessels in Figure 3, $n = 6$. C) Count of osterix+ osteoprogenitor cells in Figure 3, $n = 6$. D – F) Count of ALP+, OPN+, and Osterix+ cells in Figure 8, $n = 6$. G) Immunofluorescence images of HIF-1α expression in vitro and in vivo. H) Statistical analysis of *Vegf* mRNA expression in vivo, $n = 6$. I) Statistical analysis of *Vegf* mRNA expression in vitro, $n = 3$. All data are expressed as the mean ± SD. Statistical analysis was performed using one-way ANOVA with Tukey's multiple comparison tests. ns, no significant difference. **$p < 0.01$, ***$p < 0.001$.

Table S1.

		**Sequence (5'->3')**	**species**
Cthrc1	Forward primer	TCGCTTCGGCTCAAATGCAGGA	Rat
	Reverse primer	CACCAATCCCTTCACAGAGTCCTTCCA	Rat
Trap	Forward primer	TCCCCAATGCCCCATTCCACA	Rat
	Reverse primer	TGCCGAGACATTGCCAAGGTGA	Rat
Nfatc1	Forward primer	CAAAGGGCAGCATCCGAAAGTGGTT	Rat
	Reverse primer	AAACGCACAATCCACAGACCGCGTA	Rat
Ctsk	Forward primer	ATGTGAACCATGCCGTGTTGGTGGT	Rat
	Reverse primer	AAAGCCACCGTTCGCTTGCACTGTT	Rat
C-fos	Forward primer	AGCATCGGCAGAAGGGGCAAAGTA	Rat
	Reverse primer	TTGGCAATCTCGGTCTGCAACG	Rat
Emcn	Forward primer	CCAAAAACCAAAGACGACCGAGACG	Rat
	Reverse primer	AGCGCGATAACCACGGGCAAAA	Rat
Tgfβ1	Forward primer	TGCTAATGGTGGACCGCAACAACG	Rat
	Reverse primer	TTGAATCTCTGCAGGCGCAGCTCT	Rat
Tgfβ3	Forward primer	CCAAGCCAAAGTCCCCTGGAATTG	Rat
	Reverse primer	TGACCTCTGCCTTTGAGTCCAGCA	Rat
Pdgfa	Forward primer	TGAGGAAGCCATTCCCGCAGTTTG	Rat
	Reverse primer	TGGCACTTGACGCTGCTGGTGTTA	Rat
Pdgfb	Forward primer	TGAACATGACCCGAGCACATTCTGG	Rat
	Reverse primer	TTGGCATTGGTGCGATCGATGA	Rat
Fgf1	Forward primer	AGTACTTGGCCATGGACACCGAAG	Rat
	Reverse primer	TTCTTCTTGAGGCCCACAAACCAG	Rat
Alp	Forward primer	CCCAAGAGACCTTGAAAAATGCCCTGA	Rat
	Reverse primer	TGTCACTGTGGAGACGCCCATACCAT	Rat
Ocn	Forward primer	TTGTGGTGGTGGTAGGGGTTGGAGAA	Rat
	Reverse primer	TCTGGTGCGATCCATCACAGAGCA	Rat
Col1	Forward primer	CCAACGACGTCGAACTTGTTGCTGA	Rat
	Reverse primer	CAATGTCAAGGAATGGCAGGCGAGA	Rat

Opn	Forward primer	TTGGCTGAAGCCTGACCCATCTCA	Rat
	Reverse primer	TCGTCATCATCGTCCATGTGGTCA	Rat
Osterix	Forward primer	TGCAGCAAGTTTGGTGGCTCCAG	Rat
	Reverse primer	GGAGGGGAGACCATTGGTGCTTGA	Rat
Vegf	Forward primer	CCCACGACAGAAGGGGAGCAGAAA	Rat
	Reverse primer	TCATTGCAGCAGCCCGCACA	Rat
Si-Cthrc1	sequence	GCTTCTACTGGGTGGAATTCT	Rat
Si-Cthrc1 Fluorescent control	sequence	GCTTCTACTGGGTGGAATTCT-cy5	Rat
Si-Cthrc1 negative control	sequence	CCTCCTTTGGGGTGGAATATT	Rat
